# Extensive Diversity of Viruses in Millipedes Collected in the Dong Nai Biosphere Reserve (Vietnam)

**DOI:** 10.3390/v16091486

**Published:** 2024-09-19

**Authors:** Alexander G. Litov, Irina I. Semenyuk, Oxana A. Belova, Alexandra E. Polienko, Nguyen Van Thinh, Galina G. Karganova, Alexei V. Tiunov

**Affiliations:** 1 Laboratory of Biology of Arboviruses, FSASI Chumakov Federal Scientific Center for Research and Development of Immune-and-Biological Products of RAS, 108819 Moscow, Russia; mikasusha@bk.ru (O.A.B.); polienko.ae@yandex.ru (A.E.P.); karganova@bk.ru (G.G.K.); 2 Institute for Translational Medicine and Biotechnology, Sechenov University, 119991 Moscow, Russia; 3 A.N. Severtsov Institute of Ecology and Evolution, 119071 Moscow, Russia; free-cat@bk.ru (I.I.S.); a_tiunov@mail.ru (A.V.T.); 4 Southern Branch, Russian–Vietnamese Tropical Scientific and Technological Center, Ho Chi Minh City 70001, Vietnam; thinh39b@gmail.com

**Keywords:** tropical forest, Cat Tien National Park, *Secoviridae*, *Nairoviridae*, *Xinmoviridae*, *Picornavirales*, *Alternaviridae*, *Mitoviridae*, zhaoviruses, *Qinviridae*

## Abstract

Advances in sequencing technologies and bioinformatics have led to breakthroughs in the study of virus biodiversity. Millipedes (Diplopoda, Myriapoda, Arthropoda) include more than 12,000 extant species, yet data on virus diversity in Diplopoda are scarce. This study aimed to explore the virome of the millipedes collected in the Dong Nai Biosphere Reserve in Vietnam. We studied 14 species of millipedes and managed to assemble and annotate the complete coding genomes of 16 novel viruses, the partial coding genomes of 10 more viruses, and several fragmented viral sequences, which may indicate the presence of about 54 more viruses in the studied samples. Among the complete and partial genomes, 27% were putative members of the order *Picornavirales*. Most of the discovered viruses were very distant from the viruses currently present in the relevant databases. At least eight viruses meet the criteria to be recognized as a new species by the International Committee on Taxonomy of Viruses, and, for two of them, a higher taxonomic status (genus and even family) can be suggested.

## 1. Introduction

The use of high-performance metagenomic technologies has facilitated breakthroughs in the study of viruses [1,2,3]. This approach has significantly expanded the known diversity of arthropod viruses, had a significant impact on viral taxonomy [2,4,5], and shown that studying the viromes of neglected phyla of organisms improves our understanding of viral evolution and ecology. Among arthropods, bloodsucking arthropods such as mosquitoes and ticks are the most thoroughly investigated [6,7,8,9,10] due to their potential to transmit pathogens to humans or domesticated animals. Some economically important arthropods [11,12,13,14] are also being actively studied. Research on other groups is comparatively rare but has led to a significant increase in knowledge of virus biodiversity.

Millipedes constitute the third-largest class of terrestrial arthropods (Class Diplopoda, sub-phylum Myriapoda, phylum Arthropoda) with more than 12,000 recognized species, which are more diverse in tropical areas [15,16]. Millipedes are mainly saprotrophic animals that feed on dead plant material such as leaf litter, fallen fruits, etc., but sometimes they become pests and feed on crops, consuming seedlings or tubers [17,18]. In cultivated and urban areas, millipedes are abundant in green patches and are especially attracted to compost spots; there are also a number of synanthropic species with a worldwide distribution [19,20]. While millipedes usually do not interfere with humans directly, they can cause problems due to occasional outbreaks in abundance or by transmitting certain bacterial pathogens [21,22,23].

In Southeast Asia, millipedes play an important role in forest ecosystems; their local fauna can reach more than 40 species [24]. Some species prefer pristine forest habitats (such as *Cryxus ovalis* and *Hylomus cattienensis*, used in this research), while others are tolerant to highly disturbed ecosystems (*Antheromorpha festiva* and *Thyropygus carli*); additionally, some inhabit urban areas and are synanthropic (*Trigoniulus corallinus* and *Atopochetus dollfusii*). The last two species, especially *T. corallinus*, often come into contact with people as they have a pantropical distribution and often appear in greenhouses in colder areas. This species is also used to obtain millicompost for organic farming purposes [25]. In addition to having a very wide distribution, *A. dollfusii*, known by the common name Thai Rainbow Millipede, is very popular among keepers and breeders; cultures of this species are maintained with the frequent influx of specimens from the wild. 

To our knowledge, no work specifically dedicated to characterizing millipede viromes exists. However, novel viruses have been found in millipedes in large-scale metagenomic studies. A few dozen new viruses were identified in Myriapoda mixes during a large-scale arthropod virome study in China [1]. The cress-like millipede associated circular virus 1 was identified in the *Oxidus* sp. collected in New Hampshire, USA, during a study dedicated to screening for cress-like viruses in terrestrial arthropods [26]. A millipede associated circo-like virus was also detected in *Epanerchodus kimi* during a study on the presence of circoviruses in Korean bats [27]. A large-scale in silico search of the mitoviruses yielded a new virus in *Glomeridella minima* from Austria [28].

In this study, we explore the biodiversity of viruses in the metagenomes of the millipedes found in the Dong Nai (Cat Tien) Biosphere Reserve (Vietnam). In total, we studied 14 species of millipedes belonging to 13 genera and were able to assemble and annotate 16 complete, and 10 partial genomes of novel viruses. Several more dozen short sequences with homology to the viral proteins were detected, which may indicate the presence of about 54 more viruses.

## 2. Materials and Methods

### 2.1. Collection, Homogenization, and Pooling of Millipedes

Millipedes were collected in the lowland monsoon tropical forest in the Nam Cat Tien sector of the Dong Nai Biosphere Reserve (11°21′–11°48′ N; 107°10′–107°34′ E) in November and December of 2022. More information about the climate, forest types, and millipede community is given in [29,30].

Millipedes were searched for visually in their typical habitats, including the forest floor, tree trunk surfaces, decaying wood, etc. In addition to forest habitats, the nearby village Nam Cat Tien (Tan Phu district, Dong Nai province) was also investigated for millipedes. Examples of the millipede species used in the study are presented in Figure S1. 

After collection, specimens were visually identified to the species level (for one animal, only the genus level of identification was possible as they are potentially undescribed species). Subsequently, the millipedes were washed in 70% ethanol, then twice in distilled water, and they were then submerged in RNA preservation buffer (sterile 60% glycerol, sterile 40% phosphate-buffered saline pH 7.4 (Sigma, St. Louis, MO, USA), 1000 units/mL of penicillin, 1000 µg/mL of streptomycin, and 2.5 µg/mL of Amphotericin B) and transported to the lab at −20 °C, where they were stored at −70 °C.

Before homogenization, the RNA storage buffer was removed and individual millipedes were washed twice in distilled water. The millipede heads and bodies were dissected and homogenized separately. Body parts of 1 cm in length or the whole body (for small specimens) were homogenized. Homogenization was performed in saline buffer using a Tissue Lyser 2 (Qiagen, Hilden, Germany) for 15 min at frequency 25 s^−1^. The amount of saline buffer used ranged from 150 μL to 500 μL depending on the specimen size. Further details about millipede homogenization are available in Table S1. Homogenates of conspecific specimens were pooled in equal amounts. The millipede species used in the study and the composition of each pool are presented in Table 1.

### 2.2. High-Throughput Sequencing Sample Preparation

RNA extraction and rRNA depletion from samples were performed as described elsewhere [31]. After sample preparation, library preparation and sequencing on the HiSeq1500 (Illumina, San Diego, CA, USA) were performed as described earlier [31]. Obtained raw sequencing reads were deposited in the SRA database (BioProject ID: PRJNA1138258).

### 2.3. Virus Discovery Pipeline

Adapter sequences, bases of low quality (<Q30) and short reads (length < 35), and 3 leading and trailing nucleotides were trimmed using Trimmomatic v0.39 [32]. The trimmed reads were de novo assembled into contigs with SPAdes v3.13.0 using the “-rnaviral” pipeline [33]. Obtained contigs were filtered using a two-stage approach.

First, the contigs containing no ORFs or ORFs with a large number of low-complexity regions (≥25% of ORF) were filtered using the seg program [34] and a custom Python script. Second, all contigs with high-confidence non-viral hits (E-value of 10^−15^ and lower), hits to known human pathogens and hits to phage phiX174 in megablast were filtered out using BLAST v.2.15.0 [35] with the nt database.

After filtering, the contigs with putative virus sequences (E-value of 0.01 or lower) were extracted using blastn in BLAST v.2.15.0 [35] with the nt_viruses database, as well as the 50 longest contigs. For this set of contigs, protein sequences from the longest ORFs (the two longest from contigs of 1000 nt and larger) were extracted and blastp was performed using BLAST v.2.15.0 and the nr database. All the sequences with virus-related hits were manually examined to determine the completeness of their genomes. In some cases, SeqMan v.7.0.0 (DNAstar Inc., Madison, WI, USA) was used (with contigs as an input) or UGENE v50.0 [36] (with raw reads as an input with up to a 10% mismatch per read allowed and “align reverse complement reads” enabled) to produce better virus assembly.

### 2.4. Estimation of Virus Assembly Quality and Virus Naming

After assembly, completeness of the virus genome was estimated manually by comparing the ORF layout with closest relatives according to blastp analysis and related viruses that complete coding genome according to ICTV. The virus was considered a complete coding sequence when it had layout similar to already known viruses, all ORFs had start and stop codons, and the start codon position was similar to closely related viruses during amino acid alignment. In some cases, when viruses were too divergent to properly estimate the start codon position, the virus was considered a complete coding genome if all other criteria were met. Segmented viruses were considered a complete coding genome when all their segments were discovered and met complete coding criteria.

Genomes were considered partial when the gaps estimated by aligning ORFs to close relatives were less than 20% of the complete coding genome. In other cases, a virus-related sequence was considered a genome fragment. Segmented viruses were considered to have a partial genome when they had one complete coding segment, even if all other segments were not identified in the sample.

Viruses with complete coding and partial genomes were named after performing a phylogenetic analysis. The name of the virus consisted of the name of the national park where the study took place (Cat Tien), or nearby rivers; genus of the millipede (unless the virus was likely not infecting millipede, see Discussion); and the virus systematic unit. For viruses unclassified to a genus level in our analysis, and in cases when the viral family consists of a single genus, the suffix “-like” was used. 

### 2.5. Data Analysis and Visualization

Total virus abundance in the sample was estimated using Bowtie 2 v.2.3.5.1 [37] by aligning reads on an index containing all the virus sequences found in the pool. The reported percentage of uniquely aligned reads was considered the percentage of viral reads in the sample. Individual virus abundance was estimated using coverage values provided by SPAdes v3.13.0 [33] after assembly. For viruses with coverage higher than 100, accurate abundance was estimated using Bowtie 2 v.2.3.5.1.

For the phylogenetic analysis, either the RNA-dependent polymerase, polyprotein, or glycoprotein (if necessary) amino acid sequence was translated and extracted from the viral contigs. Homologs of each extracted sequence were retrieved from the nr database, performing online blastp searches with the default parameters. If necessary, homolog sequences from species recognized by the International Committee on Taxonomy of Viruses (ICTV) were added to the analysis. 

The obtained sequences were aligned using MAFFT v7.310 using the E-INS-i algorithm with 1000 cycles of iterative refinement [38]. Ambiguously aligned regions were removed from alignments using TrimAL v1.4. rev 15 with automated region detection [39]. Subsequently, sequences with high gap or unknown amino acid content were removed from the alignments using a custom Python script. Maximum likelihood phylogenetic trees were constructed using IQ-TREE v.2.3.2 [40] with 1000 bootstrap replicates and automatic model detection [41]. Further details about each phylogenetic tree are available in Table S2. The obtained phylogenetic trees were visualized using FigTree v1.4.4.

Virus genomes were manually annotated and visualized using the GenomeDrawing tool (https://github.com/justNo4b/GenomeDrawing, accessed on 16 July 2024). Image post-processing was performed using the GIMP v.2.10.24 program.

## 3. Results

### 3.1. High-Throughput Sequencing

**Figure 1:**
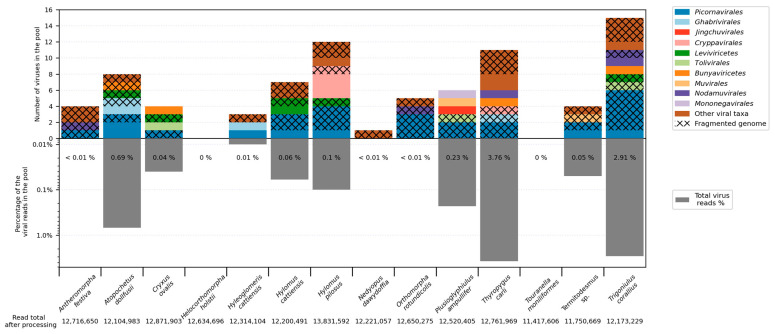
Total number of viruses (**top**) and abundance of virus-containing reads (**bottom**) in each studied pool. Distinct virus groups are marked with colors. The section is marked by crosses if only fragments of the virus genome were obtained.

The aim of the work was to describe the biodiversity of millipede viruses collected in the Dong Nai (Cat Tien) Biosphere Reserve (Vietnam). We studied fourteen pools, each composed of the members of a single millipede species. We obtained 11–13 million reads per pool (after filtering out low-quality reads) and managed to assemble and annotate the complete coding genomes of 16 novel viruses (Figure 1, Table S3). In ten cases, we were able to assemble partial coding sequences (see Section 2.4). All viruses with complete and partial sequences had relatively low amino acid identity to the viruses currently available in the GenBank databases. In several cases, low query cover was observed during blastp analysis (Table S3).

Additionally, we detected several dozen sequences with homology to the viral proteins, which may indicate the presence of about 54 more viruses (Table S4) in the studied samples (see Section 3.13). A large number of viruses detected in the study were partial or presented in the form of fragmented sequences. This can be explained by the relatively low sequencing depth (11–13 reads after filtering). It could also have prevented us from discovering additional low prevalence viruses. 

Some sequences with homology to the viral proteins had very unusual structures for viruses and thus were considered virus-like elements (see Section 3.12). 

### 3.2. Picornavirales

**Figure 2:**
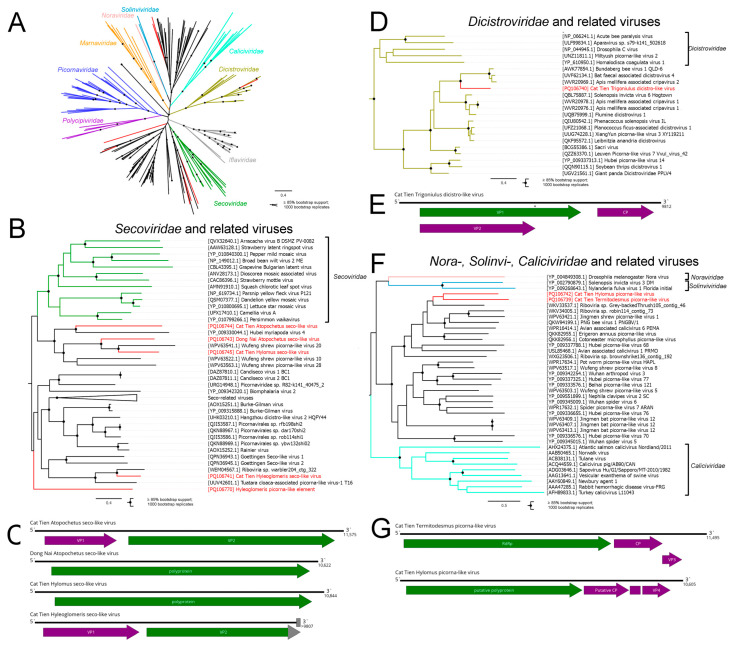
Genomic structure and phylogenetic relationships of discovered picorna-like viruses. (**A**) Unrooted phylogenetic tree of the classical members of *Picornavirales*, discovered picorna-like viruses and related viruses. The tree was constructed using the amino acid sequences of the RdRp-encoding polyprotein, with 1000 bootstrap replicates. Nodes with ≥85% bootstrap support are indicated. The scale bar represents the number of amino acid substitutions per site. Discovered viruses are shown in red. Phylogenetic groups that have ICTV-recognized members are color coded. ICTV-unrecognized groups are shown in black. (**B**) Subtree of the phylogenetic tree pictured in (**A**), depicting members of *Secoviridae* and related viruses. (**D**) Subtree of the phylogenetic tree pictured in (**A**), depicting members of *Dicistroviridae* and related viruses. (**F**) Subtree of the phylogenetic tree in (**A**), depicting members of *Nora*-, *Solinvi*-, *Caliciviridae* and related viruses. (**C**,**E**,**G**) Genome scheme of the discovered picorna-like viruses. ORFs are shown in purple. RdRp-encoding ORFs are shown in green. An asterisk (*) above ORF signifies a putative read-through of the UGA codon. Gray blocks indicate estimated gaps. Gray arrowheads indicate the absence of the stop codon within the sequenced region.

The order *Picornavirales* was established in 2008 and contains viruses with a monopartite or bipartite positive-strand RNA genome [42]. Currently, it contains nine viral families, and a large number of viruses resembling those in the order *Picornavirales* have been discovered in the last ten years [1,42]. The number of picorna-like viruses was the highest in this study (Figure 1), and picorna-like sequences were found in all pools except those of *Helicorthomorpha holstii*, *Touranella moniliformis*, and *Nedyopus dawydoffiae*. Among the complete and partial genomes, 27% (7 out of 26) were putative members of *Picornavirales* (Figure 1 and Figure 2A). The most highly represented group were seco-like viruses (Figure 2C).

Classical members of *Secoviridae* infect dicotyledonous plants. Seven genera within this family have a bi-segmented genome, while two genera have a monopartite genome with proteins encoded in the single large polyprotein [43]. A large number of viruses with homology to the seco-like polyprotein were discovered recently, mostly in invertebrates. In some cases, seco-like viruses have a genome with two distinct ORFs, encoding structural, and non-structural proteins [1,44], which is unknown for classical members of *Secoviridae*.

We discovered several contigs with homology to seco-like proteins. In four cases, the ORF length and composition corresponded to previously discovered seco- and seco-like viruses (Figure 2C). We therefore concluded that these contigs belonged to the complete or partial genomes of the novel viruses. They were named the Cat Tien Atopochetus seco-like virus (CASV), Dong Nai Atopochetus seco-like virus (DASV), Cat Tien Hylomus seco-like virus (CHSV), and Cat Tien Hyleoglomeris seco-like virus (CHySV). For CASV, DASV, and CHSV complete coding sequences were assembled, while for CHySV, about 50 terminal amino acids in the VP2 polyprotein remain unknown. We discovered another seco-like element in the pool of *Hyleoglomeris cattienensis*. However, due to its structure, which was highly unusual for a seco-like virus ORF, we did not consider it a virus (see Section 3.12).

Phylogenetically, the seco-like viruses we discovered belonged to two different parts of the seco-like virus group. CASV, DASV, and CHSV formed a monophyletic group with Hubei myriapoda virus 4, identified in unspecified Myriapoda [1], as well as three viruses discovered in *Chodsigoa smithii* shrews in China [44]. It should be noted that viruses identified in *Chodsigoa smithii* are likely to be diet-related (see Discussion).

Interestingly, although this group is monophyletic and seems to be closely related, their genome structure varies: DASV, CHSV, and Wufeng shrew picorna-like virus 10 express genomes via a single polyprotein, while the others have two ORFs. CHySV was phylogenetically grouped together with other groups of seco-like viruses: an unnamed virus discovered in the Arctic warbler *Phylloscopus borealis* and Tuatara cloaca-associated picorna-like virus-1, discovered in a New Zealand reptile *Sphenodon punctatus* (both in cloaca swabs) [45].

DASV was one of the most abundant viruses in the study. In the *Atopochetus dollfusii* pool, where it was discovered, DASV reads represented 0.64% of the total reads after filtering (Table S5). This may indicate that DASV is actively replicating in millipedes; however, future research is needed to prove this.

The family *Dicistroviridae* unifies non-segmented viruses with linear, positive-sense RNA genomes of 8–10 kb in length, which infect arthropods. The genome contains two main non-overlapping ORFs that encode non-structural and structural protein precursors [46]. 

Here, we discovered a contig with homology to the dicistro-like proteins (Figure 2E). It followed the general dicistro-like genome plan, but only if we assume a UGA read-through mechanism for ORF1 expression. This contig had a “leaky” UGAC context, a mechanism often used by viruses for controlled protein expression, although the “stop–carry on” mechanism is much more common for *Picornavirales* [47]. Overall, we concluded that this contig represented a genome of a novel dicistro-like virus and named it the Cat Tien Trigoniulus dicistro-like virus (CTDV). CDTV formed a monophyletic group with Apis mellifera associated cripavirus 2, Bundaberg bee virus 1 (both found in *Apis mellifera* bees) [11], and bat faecal associated dicistrovirus 4, discovered in *Pteropus poliocephalus* feces (Figure 2D).

Additionally, we discovered two contigs with homology to various novel picorna-like viruses. The contigs’ ORF layouts resembled the genome layouts of some *Caliciviridae* genera, with one larger RdRp-encoding polyprotein and 2–3 additional ORFs (Figure 2G) [48]. Overall, we judged that these contigs represented the genome of two novel picorna-like viruses and named them the Cat Tien Termitodesmus picorna-like virus and Cat Tien Hylomus picorna-like virus. Phylogenetically, they were grouped together, along with an unnamed virus discovered in the bird metagenome in China (GenBank ID OQ424140), but with <85% bootstrap support.

### 3.3. Tolivirales and Nodamuvirales

Orders *Tolivirales* and *Nodamuvirales* are related and can be analyzed together when the phylogenetic placements of novel viruses are discussed [1]. Both orders contain viruses with positive-strand RNA genomes. Order *Tolivirales* includes the family *Carmotetraviridae* and the family *Tombusviridae. Carmotetraviridae* contains a single member (Providence virus) with a monopartite RNA 6.1 kb in length [49]; however, many viruses that are phylogenetically close to *Carmotetraviridae* have recently been discovered. The order *Nodamuvirales* includes the families *Nodaviridae* (bi-partite RNA viruses 3.1 kb and 1.4 kb in length) [50] and *Sinhaliviridae*. A large number of novel viruses related to *Toli-* and *Nodamuvirales* have been found using HTS in the past decade [1].

**Figure 3:**
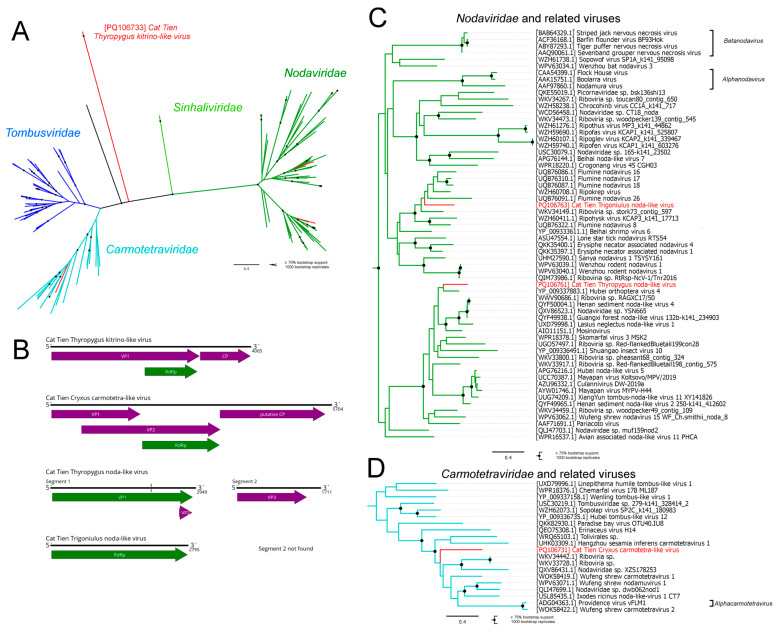
Genomic structure and phylogenetic relationships of the newly discovered toli- and noda-like viruses. (**A**) Unrooted phylogenetic tree of the classical members of *Tolivirales*, *Nodamuvirales*, discovered viruses, and related viruses. The tree was constructed using the amino acid sequences of the RdRp-encoding polyprotein, with 1000 bootstrap replicates. Nodes with ≥75% bootstrap support are indicated. The scale bar represents the number of amino acid substitutions per site. Discovered viruses are shown in red. Phylogenetic groups that have ICTV-recognized members are color coded. ICTV-unrecognized groups are shown in black. (**B**) Genome scheme of the discovered viruses. ORFs are shown in purple. RdRp-encoding ORFs are shown in green. Gray line indicates estimated gaps. (**C**) Subtree of phylogenetic tree pictured in Figure 2A, depicting members of *Nodaviridae* and related viruses. (**D**) Subtree of phylogenetic tree pictured in Figure 2A, depicting members of *Carmotetraviridae* and related viruses.

Here, we discovered several contigs with homology to *Toli-* and *Nodamuvirales* (Figure 3A). In the pool of *Thyropygus carli*, we identified two contigs with a similar structure and homology to the RdRp of some tombus-like viruses and the capsid protein (CP) of solemo-like viruses (Figure 3B). Both contigs were distantly related to known sequences from GenBank (21% aa identity in RdRp) but were closely related to each other (91% aa identity in RdRp). Thus, we deduced that the two contigs belonged to two isolates of the same virus, which we named the Cat Tien Thyropygus kitrino-like virus (CTKV). Since VP1 ORF has no homologous sequences in GenBank and its overall genome length is over 4 kb, we assume that we assembled the complete coding or at least the partial coding genome, although additional data are needed to confirm this assumption.

We were not able to confidently place CTKV in any established virus order (Figure 3A). It seems that the phylogenetic status of CTKV cannot be determined without constructing a phylogenetic tree with all currently known toli- and noda-like viruses, but this is beyond the scope of our study.

In the pool of *Cryxus ovalis*, we discovered a contig with homology to the carmotetra-like viruses and an ORF layout resembling that of the Providence virus, the single member of the family *Carmotetraviridae*. We determined that this contig represented a complete genome of the novel carmotetra-like virus and named it the Cat Tien Cryxus carmotetra-like virus (CCCV). The phylogenetic analysis showed CCCV groups together with other carmotetra-like viruses (Figure 3D).

Noda-like contigs were discovered in several millipede pools; however, we were not able to assemble a single complete coding genome of the noda-like virus. In the *Thyropygus carli* pool, we assembled two contigs with homology to the noda-like proteins. The first of them was 2.9 kb, with a small gap estimated to be about 12 nt long and a larger ORF with homology to the noda-like RdRp. The second one had homology to the noda-like capsid protein (CP). Thus, together, they represented a novel noda-like virus we named the Cat Tien Thyropygus noda-like virus (CTNV). In the pool of *Trigoniulus corallinus*, we discovered one contig with homology to the noda-like polymerase. We determined that this contig represented the first segment of a novel noda-like virus we named the Cat Tien Trigoniulus noda-like virus (CTrNV). However, we were not able to find a contig with homology to the second segment of noda-like viruses in the same pool. The phylogenetic analysis showed that CTNV and CTrNV were related to the different noda-like viruses discovered using the metagenomic approach (Figure 3C). 

### 3.4. Bunyaviricetes

The virus order *Bunyavirales* was first established in 2017 to accommodate related viruses with segmented, linear, single-stranded, negative-sense, or ambisense RNA genomes classified into nine families [51]. Recently, the order was split into two with the establishment of the class *Bunyaviricetes*, which contains 15 families. 

**Figure 4:**
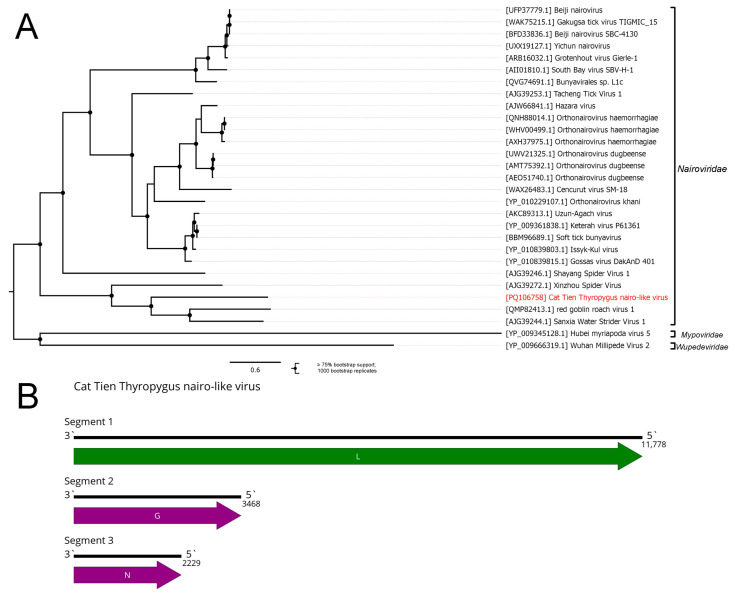
Genome and phylogenetic relationships of the Cat Tien Thyropygus nairo-like virus. (**A**) Phylogenetic tree of *Nairoviridae*, rooted on the families *Mypoviridae* and *Wupedeviridae*, used as outgroups. The tree was constructed using the amino acid sequences of the RdRp (1000 bootstrap replicates; nodes with ≥75% bootstrap support are indicated). The scale bar represents the number of amino acid substitutions per site. The discovered virus is shown in red. (**B**) Genome scheme of the Cat Tien Thyropygus nairo-like virus. ORFs are shown in purple. The RdRp-encoding ORF is shown in green.

We detected several bunya-like contigs in the studied millipedes. In the pool of *Thyropygus carli*, we discovered several contigs with homology to the various proteins of the family *Nairoviridae*. Close examination of the contigs showed that one of them contained a partial sequence of the L protein, while two others had parts of the L protein (overlapping with the major part) and full sequences of the N and G proteins, respectively. We assumed that this was likely to be an assembly artifact and manually reassembled the complete coding sequence of each protein. All the non-coding regions were deleted due to misassembly concerns, resulting in the complete coding genome of the Cat Tien Thyropygus nairo-like virus (CTNV) (Figure 4B). 

CTNV was the most abundant virus; CTNV reads represented 3.73% of the total reads after filtering (Table S5). This high number of viral reads in the sample may indicate that CTNV actively replicates in the *Thyropygus carli* millipede; however, future research is needed to prove this.

A phylogenetic analysis based on the amino acid sequences of the L protein grouped CTNV with the Xīnzhōu spider virus (*Xinspivirus xinzhouense*), red goblin roach virus 1 (*Ocetevirus paratemnopterygis*), and Sānxiá water strider virus 1 (*Striwavirus sanxiaense*) (Figure 4A) discovered in the *Neoscona nautical* spider, the *Paratemnopteryx couloniana* roach, and the *Gerridae* spp. water strider, respectively [1,2,52]. Nevertheless, according to the blastp analysis, CTNV is highly divergent even from its closest relatives (29% identity with 93% query cover compared with *Striwavirus sanxiaense*). Thus, according to the current genus demarcation criteria within the *Nairoviridae* family (less than 93% identity in the RdRp) [53], CTNV can be qualified as a new species. There are no clear numeric criteria for a genus demarcation in the *Nairoviridae* family, with multiple factors (phylogenetic relationships, genome architecture, virion antigenicity, and virus ecology) being taken into account [53]. Since CTNV is highly phylogenetically divergent and is the first nairovirus discovered in millipedes, we believe that the demarcation of a new genus for CTNV can be considered.

Additionally, we discovered several contigs with homology to bunya-like proteins. In the pool of *Trigoniulus corallinus*, we identified a contig representing a full L protein (Figure S2C) of a very distant relative of the Hubei bunya-like virus 11 (15% query cover, 26% aa identity) [1], also discovered in millipedes. Since we sequenced the complete coding sequence of the RdRp-encoding segment, we putatively named it the Cat Tien Trigoniulus phasma-like virus (CTPV). CTPV was highly abundant (2.52% of the total reads after filtering). Phylogenetically, CTPV forms a monophyletic group with Hubei bunya-like virus 11 and Beihai hermit crab virus 2 (Figure S2A). No contigs with homology to bunya-like G or N proteins were found, except for some small fragments related to phasma-like glycoprotein, with much lower coverage and quite an unorthodox size and protein layout (see Section 3.12).

In the pool of *Cryxus ovalis*, we discovered two contigs with homology to bunya-like L and G proteins (Figure S2A). However, any attempts to recover the third segment failed. Interestingly, while the L protein sequence, together with the rice phasma-like virus 1 L protein, formed a sister group to classical *Phasmaviridae*, the G protein was closer to Wuhan millipede virus 2 (*Wupedeviridae*) [2]. Since no other bunya-like sequences were found in the *Cryxus ovalis*, preliminarily, we determined that these L and G segments belonged to a single virus, which we named the Cat Tien Cryxus phasma-like virus. It is possible, however, that these two segments belong to different viruses. 

### 3.5. Mononegavirales

The order *Mononegavirales* was established in 1991 to accommodate related viruses with non-segmented, linear, single-stranded, negative-sense RNA genomes [54]. The family *Xinmoviridae* includes viruses with negative-strand RNA 9–14 kb in length, encoding three to six proteins. Members of the family have mostly been discovered in various species of insects using HTS [4]. Here, we found several contigs with homology to the xinmovirid protein sequences.

**Figure 5:**
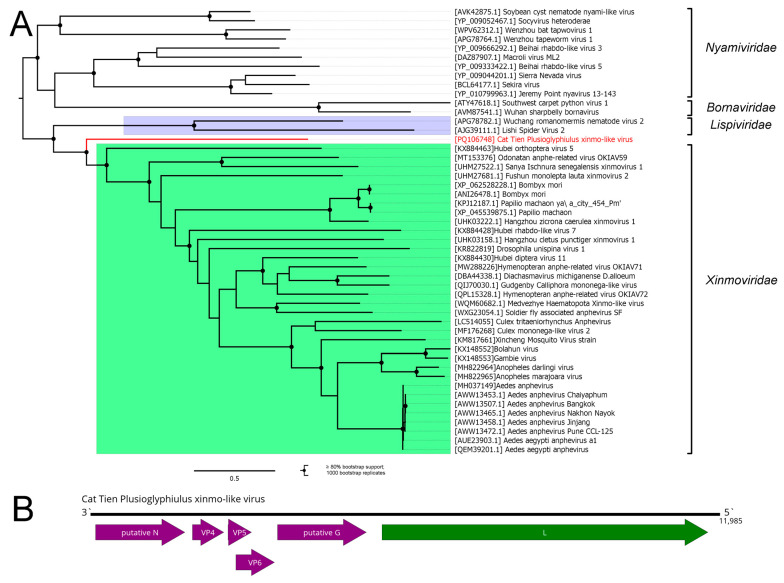
Genomic structure and phylogenetic relationships of the Cat Tien Plusioglyphiulus xinmo-like virus. (**A**) Midpoint-rooted phylogenetic tree of the Cat Tien Plusioglyphiulus xinmo-like virus. The tree was constructed using the amino acid sequences of the RdRp (1000 bootstrap replicates; nodes with ≥80% bootstrap support are marked). The scale bar represents the number of amino acid substitutions per site. The discovered virus is shown in red. Clades representing *Xinmoviridae* and *Lispiviridae* families are highlighted. (**B**) Genome scheme of the Cat Tien Plusioglyphiulus xinmo-like virus. ORFs are shown in purple. RdRp-encoding ORF is marked in green.

In the pool of *Plusioglyphiulus ampullifer*, we managed to assemble an almost 12 kb contig. It encoded 6 ORFs (Figure 5B), with the largest of them having weak homology to *Xinmoviridae*, *Lispiviridae*, *Bornaviridae*, and *Nyamiviridae* polymerases, with the closest relative (84% query cover and 26.5% identity, according to BLAST) being Odonatan anphe-related virus OKIAV59 (*Xinmoviridae; Ulegvirus freckenfeldense*). We judged this contig to represent a complete coding genome of the novel virus, and we named it the Cat Tien Plusioglyphiulus xinmo-like virus (CPXV).

The phylogenetic analysis, which was performed using the amino acid sequences of the RdRp of the CPXV, as well as some members of the families *Xinmoviridae*, *Lispiviridae*, *Bornaviridae*, and *Nyamiviridae*, placed CPXV outside of the abovementioned families (Figure 5A). It was closest to and formed a monophyletic group with members of the *Xinmoviridae* family, while at the same time being the most basal in that group. According to the current genus demarcation criteria within the *Xinmoviridae* family (less than 60% identity in the RdRp) [4], CPXV is qualified to be a new genus.

It should be noted that CPXV was isolated from millipedes, while all the other members of the family *Xinmoviridae*, including related unclassified viruses, were isolated from insects (at least in cases where the isolation source was known) [4]. Combined with CPXV’s phylogenetic placement, genome structure, and high divergence in RdRp, the creation of a separate family for CPXV can be considered.

### 3.6. Ghabrivirales

**Figure 6:**
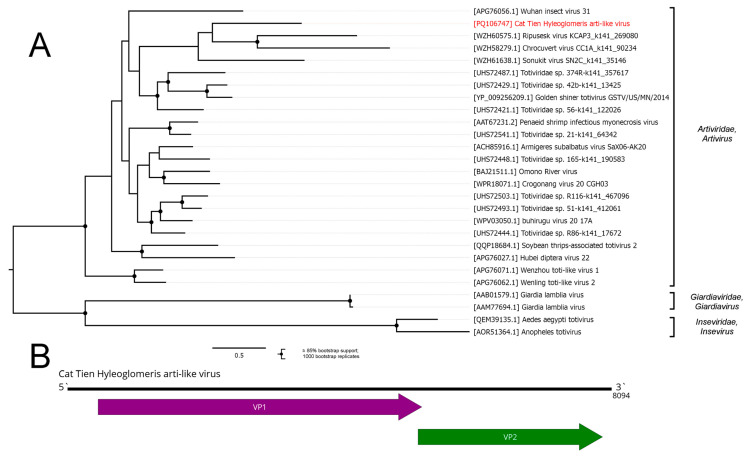
Genomic structure and phylogenetic relationships of the Cat Tien Hyleoglomeris arti-like virus. (**A**) Phylogenetic tree of the family *Artiviridae*, rooted on the families *Giardiviridae* and *Inseviridae*, used as outgroups. The tree was constructed using the amino acid sequences of the RdRp (1000 bootstrap replicates; nodes with ≥85% bootstrap support are marked). The scale bar represents the number of amino acid substitutions per site. The discovered virus is marked in red. (**B**) Genome scheme of the Cat Tien Hyleoglomeris arti-like virus. ORFs are shown in purple. The RdRp-encoding ORF is marked in green.

The order *Ghabrivirales* was established by the ICTV in 2020 and, at that time, included four families of dsRNA viruses: *Chrysoviridae*, *Megabirnaviridae*, *Quadriviridae*, and *Totiviridae.* However, a large number of highly diverse viruses that contain *Ghabrivirales*-related polymerase and genome structures generally resembling classical *Ghabrivirales* members (“toti-like viruses”) have been detected in the past few years. This led to the reorganization of the order *Ghabrivirales* in 2024, with 15 new families and 12 new genera established [55]. Here, we discovered several contigs with homology to the *Ghabrivirales*-related proteins. In the pool of *Hyleoglomeris cattienensis*, we discovered a contig with homology to the *Artiviridae* polymerases and a gene organization that is typical for *Artiviridae* members (Figure 6B). It had 38% identity in the RdRp to the closest relative according to the BLAST analysis; thus, we decided that it is a novel virus and named it the Cat Tien Hyleoglomeris arti-like virus (CHAV). 

According to the phylogenetic tree constructed using the amino acid sequences of the RdRp, CHAV was confidently grouped together with other members of the *Artivirus* genus. During the reorganization of *Ghabrivirales*, <70% amino acid sequence identity was established as a species demarcation criterion; thus, CHAV, with only 38% homology to the closest relative, qualifies as a new species in the *Artivirus* genus.

**Figure 7:**
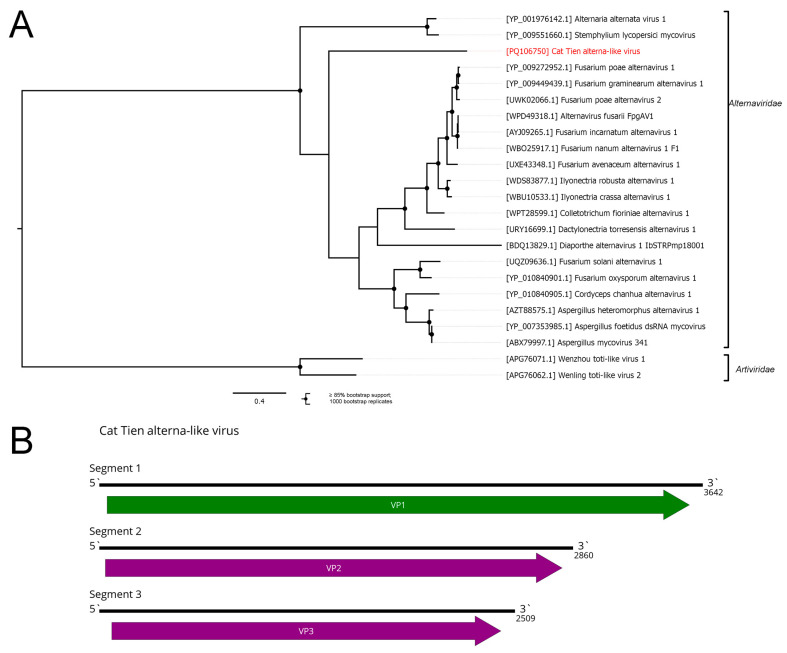
Genomic structure and phylogenetic relationships of the Cat Tien alterna-like virus. (**A**) Phylogenetic tree of the family *Alternaviridae*. The tree was constructed using the amino acid sequences of the RdRp (1000 bootstrap replicates; nodes with ≥85% bootstrap support are indicated), and rooted on the family *Artiviridae*, used as an outgroup. The scale bar represents the number of amino acid substitutions per site. The discovered virus is marked in red. (**B**) Genome scheme of the Cat Tien alterna-like virus. ORFs are shown in purple. The RdRp-encoding ORF is marked in green.

Additionally, we discovered contigs with homology to the various *Alternaviridae* proteins in the pool of *Atopochetus dollfusii*. The *Alternaviridae* family was established in 2022 with a single genus, *Alternavirus*, and contains dsRNA viruses of fungi with three to four segments [56]. In the *Atopochetus dollfusii* pool, we were able to find three contigs containing ORFs with homology to the first, second, and third segments’ ORFs of *Alternaviridae* members (Figure 7B). All three segments were highly divergent from their closest relatives. We were not able to find any sequences with homology to the fourth segment of the alternaviruses; however, we cannot exclude the possibility that the fourth segment is too divergent to be identified via a BLAST search. Nevertheless, because some members of *Alternaviridae* contain three segments, we decided to consider these three segments as full coding genomes of the novel virus and named it the Cat Tien alterna-like virus (CAV). 

According to the phylogenetic tree (Figure 7A), CAV is grouped together with other members of family *Alternaviridae*. According to the phylogenetic placement, it is likely that CAV infects unknown species of fungi (likely consumed by *Atopochetus dollfusii*), rather than the millipede itself. CAV divergence in terms of the RdRp from its closest relative (less than 70% identity), as well as in other proteins, and the absence of a fourth segment, suggest that it can be proposed as a novel species within the genus *Alternavirus.*

### 3.7. Cryppavirales

The order *Cryppavirales*, with a single family, *Mitoviridae*, includes positive-sense RNA viruses from 2.1 to 4.4 kb, encoding a single protein (RdRp) [57,58]. These viruses reproduce in the mitochondria of fungi, plants, and invertebrates [28,59], and many of them are known to use UGA codons that encode Trp [58]. 

**Figure 8:**
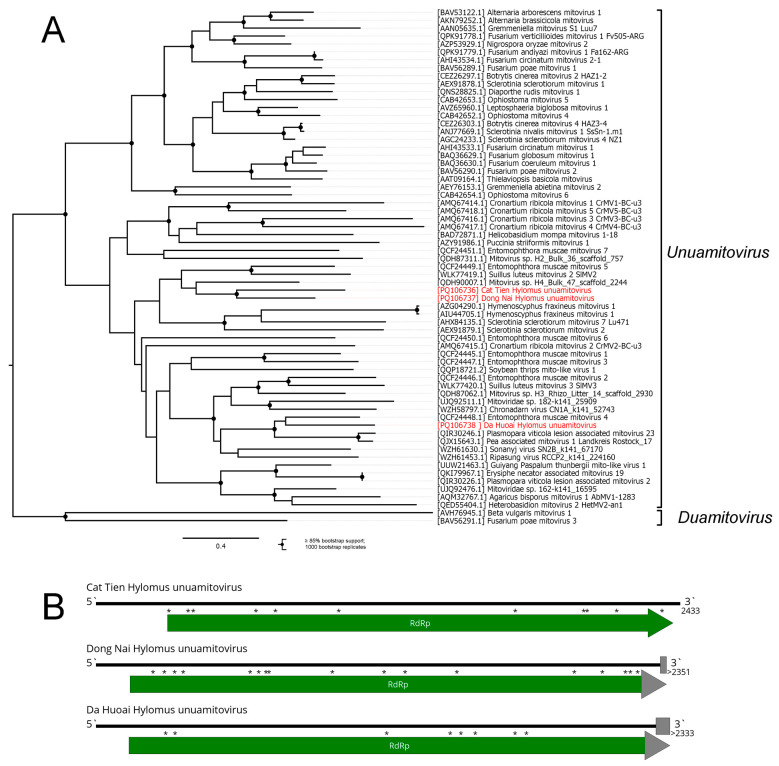
Genomic structure and phylogenetic relationships of discovered unuamitoviruses (**A**) Phylogenetic tree of the genus *Unuamitovirus*, rooted on the genus *Duamitovirus*, used as an outgroup. The tree was constructed using the amino acid sequences of the RdRp (1000 bootstrap replicates; nodes with ≥85% bootstrap support are indicated). The scale bar represents the number of amino acid substitutions per site. Discovered viruses are marked in red. (**B**) Scheme of the discovered unuamitoviruses genomes. The RdRp-encoding ORF is marked in green. Each * above an ORF signifies a UGA codon that encodes Trp. Gray blocks indicate estimated gaps. Gray arrowheads indicate the absence of the stop codon within the sequenced region.

Here, we found three contigs with homology to the mitoviruses in the pool of *Hylomus pilosus*. The contigs followed the genome scheme of the genus *Unuamitovirus*, with a single RdRp-encoding ORF that can be properly translated only when using the invertebrate mitochondrial genetic code, with 8–17 UGA codons within the RdRp-encoding sequence (Figure 8B). Additionally, alignment showed that it is likely that a non-standard start codon (UUG) should be used for all of the contigs as well; this is sometimes used in the mitochondrial genomes of invertebrates, including millipedes [60,61]. 

Since all the contigs were divergent from each other (Figure 8A), we decided that they represented three distinct viruses: the Cat Tien Hylomus unuamitovirus (CHUV), Dong Nai Hylomus unuamitovirus (DNHUV), and Da Huoai Hylomus unuamitovirus (DHHUV). 

When compared with their closest relatives, all three viruses had low identity in terms of the RdRp, according to BLAST (33%, 38%, and 51%, respectively). Phylogenetically, all of them can confidently be grouped with other *Unuamitovirus* members (Figure 8A). For DNHUV and DHHUV, we assembled only partial genomes, with no stop codon in the RdRp ORF (Figure 8B). However, according to the alignments, the estimated unsequenced ORF length was rather small (24 and 57 nt for DNHUV and DHHUV, respectively). However, in some cases, unusual stop codons were used in the mitochondria coding sequences [61], and there was a chance that the presented DNHUV and DHHUV genomes were complete.

For CHUV, the full coding sequence was determined; thus, it could be a subject for classification by ICTV. In the most recently accepted *Mitoviridae* proposal, <70% RdRP amino acid sequence identity was established as a species demarcation criterion [62]; thus, CHUV, with only 33% homology to its closest relative, qualifies as a new species in the *Unuamitovirus* genus.

### 3.8. Jingchuvirales

The order *Jingchuvirales* incorporates negative-sense RNA viruses with genomes of 9.1–15.3 kb in length. Their genomes can be represented by a single RNA molecule or divided into two segments, and, in both cases, the genome can be linear or circular [63]. 

**Figure 9:**
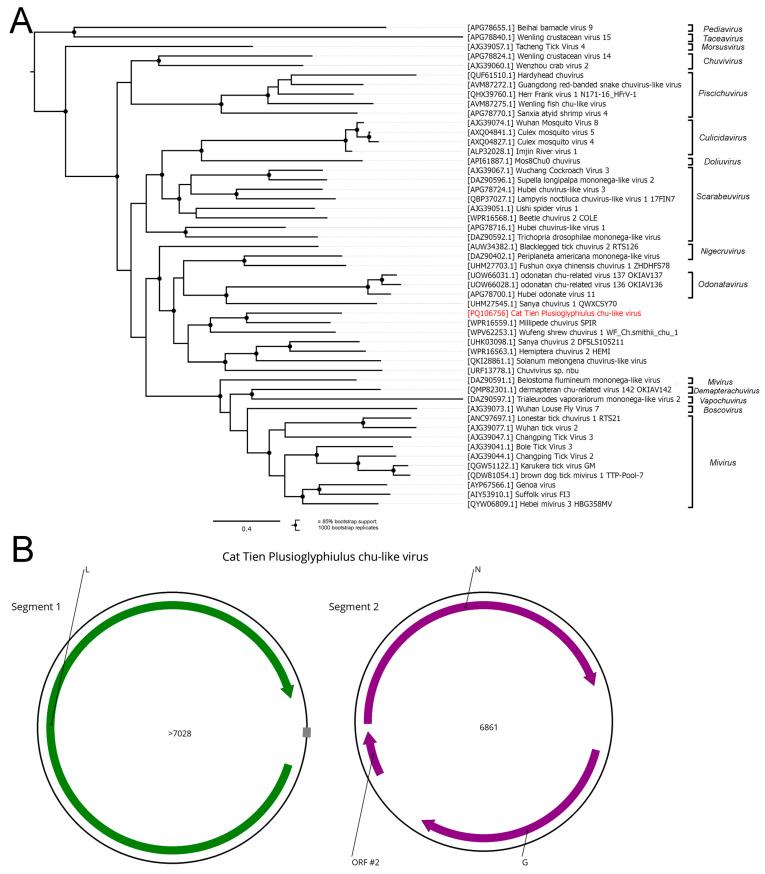
Genomic structure and phylogenetic relationships of the Cat Tien Plusioglyphiulus chu-like virus (**A**) Midpoint-rooted phylogenetic tree of the family *Chuviridae*. The tree was constructed using the amino acid sequences of the RdRp (1000 bootstrap replicates; nodes with ≥85% bootstrap support are indicated). The scale bar represents the number of amino acid substitutions per site. The Cat Tien Plusioglyphiulus chu-like virus is marked in red. (**B**) Scheme of the Cat Tien Plusioglyphiulus chu-like virus genome. ORFs are shown in purple. The RdRp-encoding ORF is marked in green. The gray block indicates the estimated gap.

Here, we found two contigs with homology to the family *Chuviridae* in the pool of *Plusioglyphiulus ampullifer*. One of the contigs had ORFs with homology to the G and N proteins of *Chuviridae* as well as one additional minor ORF, and direct nucleotide repeats on the ends. Since it is known that *Chuviridae* members can have a circular genome, we assumed that this repeat structure was an artifact of the read assembly and deleted one of them to form a complete second segment of the virus. The second contig encoded a single ORF with homology to the *Chuviridae* members L protein, but it did not have any direct repeats. We determined that these contigs represented the complete coding sequence of the first segment of the virus. Thus, both of these contigs represented two circular segments of the novel chu-like virus (Figure 9B), named the Cat Tien Plusioglyphiulus chu-like virus (CPCV).

A phylogenetic analysis based on the amino acid sequence of L proteins (Figure 9A) showed that CPCV forms a monophyletic group with the millipede chuvirus, discovered in *Spirostreptida* spp. [64], and the Wufeng shrew chuvirus 1, discovered in the *Chodsigoa smithii* shrew [44]. CPCV was quite distant from its closest relative (45% identity with 99% query cover). According to the current species demarcation criteria within the *Chuviridae* family (less than 90% amino acid identity RdRp) [63], CPCV is qualified to be a new species.

### 3.9. Zhaoviruses 

Zhaoviruses are a group of novel viruses, first proposed by Shi and colleagues [1], but this group is still unrecognized by the ICTV. This group is so divergent that it can be considered a new virus family or even order. At the time of discovery, all of the members except for Changjiang zhaovirus-like virus 1 produced viable proteins only with the usage of the ciliate, dasycladacean, and hexamita translation table, leading to the suggestion that this group might infect protists [1]. Zhaoviruses’ RdRps show significant similarities to the RdRps of the Cilio virus and Brinovirus that were isolated from wastewater and also use the ciliate translation table [65]. 

**Figure 10:**
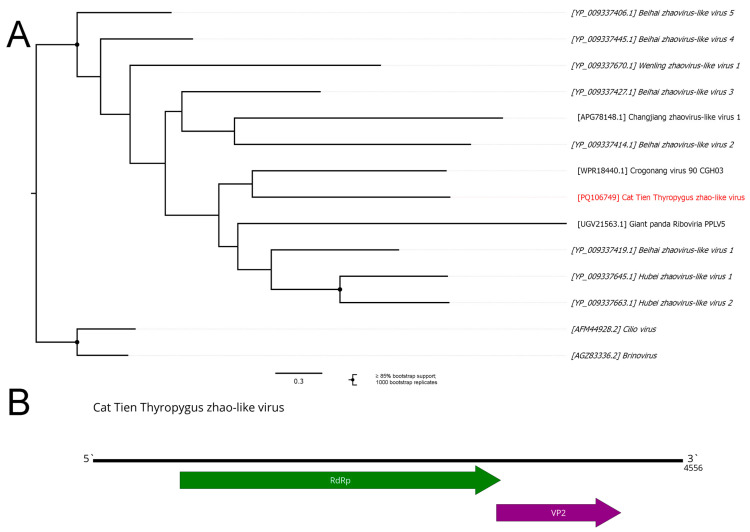
Genomic structure and phylogenetic relationship of zhao-like viruses. (**A**) Phylogenetic tree of zhaoviruses, rooted in the Cilio–Brinovirus clade. The tree was constructed using the amino acid sequences of the RdRp (1000 bootstrap replicates; nodes with ≥85% bootstrap support are marked). The scale bar represents the number of amino acid substitutions per site. The discovered virus is marked in red. Viruses using the ciliate translation table are italicized. (**B**) Scheme of the Cat Tien Thyropygus zhao-like virus genome. ORFs are shown in purple. The RdRp-encoding ORF is marked in green.

We discovered a 4556 nt contig containing two ORFs. One of them had homology to zhao-like polymerases; another one had slight homology to the CP of some toli-like viruses (Figure 10B). Thus, we concluded that this contig belonged to the genome of the novel zhao-like virus and named it the Cat Tien Thyropygus zhao-like virus (CTZV). Overall, the genome appeared to be complete (Figure 10B), and it could be translated using the standard genetic code. However, if we assume a UGA read-through or the usage of genetic code that allows for UGA translation, the genome can be considered partial, but this question cannot be resolved without a molecular biological study.

Phylogenetically, CTZV is grouped with other zhao-like viruses (Figure 10A). It was close (although with low branch support) to Crogonang virus 90, discovered in the *Gonidea angulate* shellfish hemolymph [66]. Notably, the Crogonang virus 90 genome can also be translated using standard genetic code. 

### 3.10. Muvirales

The order *Muvirales* includes a single family, *Qinviridae*, with only one genus, *Yingvirus*. Members of this family have bi-segmented negative-sense RNA genomes. The first segment is 5.6–6.6 kb in length and encodes a large ORF that encodes RdRp. The second one is much smaller (1.6–1.9 kb) and encodes nucleocapsid protein [5].

**Figure 11:**
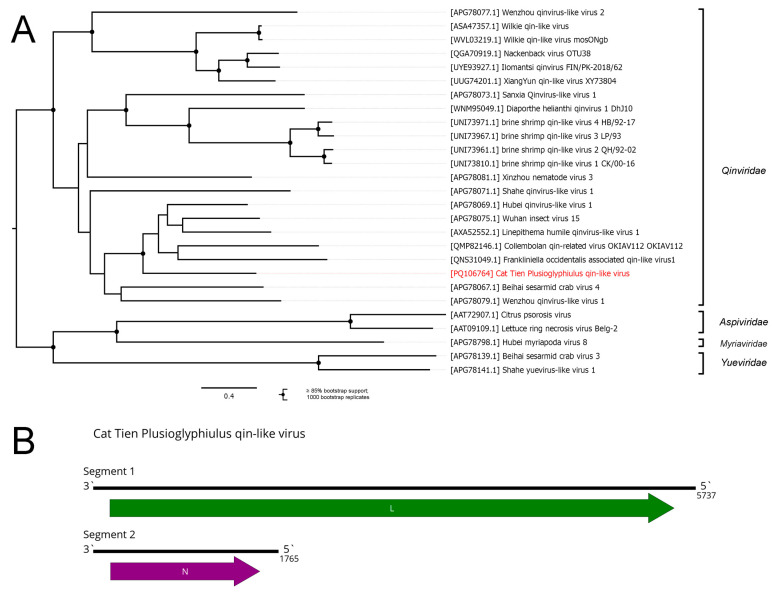
Genomic structure and phylogenetic relationships of the Cat Tien Plusioglyphiulus quin-like virus. (**A**) Phylogenetic tree of the family *Qinviridae*, rooted on members of the families *Yueviridae*, *Myriaviridae*, and *Aspiviridae*, used as outgroups. The tree was constructed using the amino acid sequences of the RdRp (1000 bootstrap replicates; nodes with ≥85% bootstrap support are indicated). The scale bar represents the number of amino acid substitutions per site. The discovered virus is marked in red. (**B**) Scheme of the Cat Tien Plusioglyphiulus quin-like virus genome. ORFs are shown in purple. The RdRp-encoding ORF is marked in green.

Here, we discovered two contigs in the pool of *Plusioglyphiulus ampullifer*. One of them had homology to the *Qinviridae* RdRp, and the second one to the N protein (Figure 11B). We decided that these two contigs represented a genome of a single virus, and we named it the Cat Tien Plusioglyphiulus qin-like virus (CPQV).

According to the phylogenetic tree based on the aa sequences of the *Qinviridae* RdRps, CPQV was confidently placed within the *Qinviridae* family. In particular, it formed a well-supported group with the previously described viruses of *Frankliniella occidentalis* trips [67], *Anurida maritima* springtails [52], *Linepithema humile* ants [68], and unspecified insects and some Diptera [1]. 

CPQV is highly divergent from all other members of *Qinviridae*, with only 35% identity in terms of the RdRp (compared to the closest relative, according to BLAST). However, no genus demarcation criterion is established for the family. *Qinviridae* species demarcation criteria require new viruses to be phylogenetically distinct based upon an analysis of the RdRp, with no numerical criterion given [5]. In our opinion, CPQV is phylogenetically distinct from other viruses (Figure 11A) and contains a complete coding sequence; thus, it can be reviewed by the ICTV as a candidate for a new species in the *Qinviridae* family.

### 3.11. Partial Virus Genomes

In addition to the abovementioned viral groups, we discovered contigs representing the partial genomes of hepe-like, solemo-like, and ashce-like viruses. 

*Ashcevirus* is one of the multiple genera of the levi-like bacteriophages (*Leviviricetes*) [69]. Here, we discovered a partial genome of the novel ashce-like virus in the pool of *Hylomus cattienensis*. The sequence was 3.6 kb length, with about 9 nt lacking on the 3′ end of the genome, resulting in the truncated sequence for the RdRp (Figure S3B). The phylogenetic analysis confidently placed this virus in the genus *Ashcevirus* (Figure S3A). It should be noted that, for *Leviviricetes*, an automatic approach to virus classification is accepted, with all genomes encoding a maturation protein with a minimum length of 350 amino acid residues, an RdRp longer than 500 aa, and less than 80% aa identity of the RdRp considered as a new species [69]. Our contig, named the Cat Tien ashcevirus (CAsV), meets all these criteria and can be reviewed by the ICTV as a candidate for a new species.

Recently, a number of viruses with homology to *Solemoviridae* polymerases were discovered in arthropods [1]. Here, we discovered a partial genome of the novel solemo-like virus. The contig was 2.8 kb in length, with at least 204 nt estimated to be lacking on the 3′ end of the genome. The second segment, usually present in solemo-like viruses, was not found. The phylogenetic analysis placed the Cat Tien Hylomus solemo-like virus together with the Beihai sobemo-like virus 23 and Shahe sobemo-like virus 1, discovered in octopuses and an unspecified freshwater arthropod, respectively [1], albeit with low bootstrap support (Figure S4A).

Members of the *Hepeviridae* family are small viruses with positive-sense RNA genomes that infect mammals, birds, and fish. Here, we found a hepe-like virus in the pool of *Trigoniulus corallinus*. We did not find a stop codon on the polyprotein-encoding ORF, thus, the genome is not coding-complete. However, due to it being larger (8 kb) than almost all of its relatives, we cannot estimate how large the gap is (Figure S5B). This virus clustered together with a virus discovered in a bird cloacal swab metagenome (Figure S5A). This virus was abundant (0.33% of total reads after filtering), suggesting that it might replicate in the millipede; however, future research is needed to prove this.

### 3.12. Virus-like Elements

**Figure 12:**
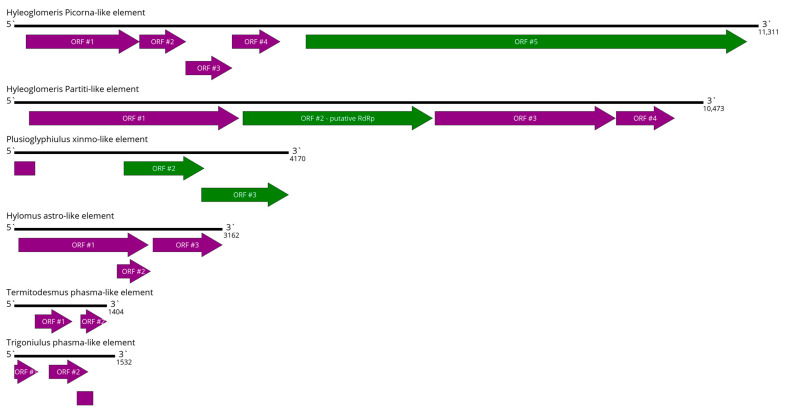
Schemes of the virus-like elements discovered in the *Hyleoglomeris cattienensis*. ORFs are shown in purple. RdRp-encoding ORFs are marked in green.

In the pool of *Hyleoglomeris cattienensis*, we discovered two virus-like elements (Figure 12). The first of them was 11,311 nt in length and had homology to a polyprotein of the picorna-like viruses (34% query cover, 30.9% identity, 9 × 10^−95^ E-value for ORF #5), including putative polymerases. However, the ORF layout was very unusual for classical members of *Secoviridae* and seco-like viruses. All of its closest relatives also use either a single polyprotein layout, which is typical for many picornaviruses, or a seco-like layout with two ORFs (Figure 2C). Thus, we cannot confidently say that this contig represented a genome of the functional virus.

Furthermore, we discovered a 10,473 nt contig with four ORFs (Figure 12). The second ORF had some homology (17% query cover, 28.4% identity, 4 × 10^−7^ E-value) to the RdRp of the Nelson Partiti-like virus 1, discovered in the common wasp *Vespula vulgaris* [70]. Two other ORFs had some vague homology to Thermoanaerobaculia bacterium sulfotransferase and Agrotis segetum nucleopolyhedrovirus A ORF-61 (E-values of 0.009 and 0.001, respectively). While this contig seems to share some homology to partiti-like RdRps, both the ORF layout of the contig and its size are vastly different from both classical partitiviruses and recently discovered partiti-like viruses. Thus, we cannot confidently say that this contig represents the genome of the functional virus.

In the pool of *Plusioglyphiulus ampullifer*, we discovered a 4170 nt contig with three ORFs (Figure 12). Two of them had very high homology to the xinmo-like RdRps (Table S6); however, the division of the RdRp into two parts is not known for members of *Xinmoviridae*.

In the pool of *Hylomus pilosus*, we discovered a 3162 nt contig with three ORFs (Figure 12). The third one had homology to the Hubei astro-like virus CP (E-value of 10^−34^), but the two other ORFs had some homology to hypothetical proteins found in invertebrates (Table S6). While it is possible that the hypothetical proteins might be viral, currently, we cannot say that this contig represents a virus.

In the pool of *Trigoniulus corallinus* and *Termitodesmus* sp., contigs with homology to phasma-like glycoproteins were found (Table S6). At the same time, the size of the discovered ORFs and their layouts on the contig (Figure 12) are too unusual to constitute a phasma-like glycoprotein. 

Several possible explanations are possible. First, these contigs may represent the full or partial genomes of highly distant viruses with very unusual gene expressions. Second, the contigs may represent virus-like sequences integrated into the cellular genome. Third, the contigs may be a result of the misassembly of short reads. A combination of the abovementioned reasons is also possible.

### 3.13. Viral Genome Fragments

In addition to the abovementioned complete and partial virus sequences, as well as viral-like elements, several dozen contigs with homology to viral proteins were found in this study. It is unclear whether these contigs represented low-abundance millipede viruses, endogenous viral elements in a host genome, or the viruses of some millipede-associated organisms (such as components of the diet or parasites). Most of these contigs are generally small (less than 1000 nt) and only distantly related to previously described viruses (Table S4). 

Nevertheless, some relatively large (estimated to be around 50–70% of the coding genome) parts were recovered. In the pool of *Hyleoglomeris cattienensis*, we discovered a 3182 nt contig with two ORFs. One of them had homology to toli-like virus polymerases. In the pools of *Hylomus cattienensis* and *Thyropygus carli*, two dicistroviral contigs (6959 and 4861 nt, respectively) were found. Notably, the contig from *Thyropygus carli* was almost identical (blastn with 95.87% identity) to the *Aparavirus* sp. isolate R115-k141 (MZ679065), found in the river sediment in Hainan, China [71]. In *Trigoniulus corallinus*, we found a 2227 nt contig with homology to the VP1 and VP2 of the noda-like viruses. 

Among the genome fragments, 39% (21 out of 54) of the distinct contig groups had homology to various *Picornavirales* members, including ones with homology to *Caliciviridae*, *Secoviridae*, *Dicistroviridae*, *Marnaviridae*, *Iflaviridae*, and *Solinviviridae* proteins. Five groups of contigs were related to the order *Durnavirales* (partiti-like or picobirna-like viruses) and the class *Leviviricetes*. Three groups of contigs were related to noda-like viruses (*Nodamuvirales*), and tombus-carmotetra-like viruses (*Tolivirales*). A few contig groups were related to *Tymovirales*, *Polymycoviridae*, *Bunyaviricetes*, *Cryppavirales*, *Martellivirales*, *Reovirales*, *Muvirales*, *Wolframvirales*, and *Quenyaviruses* (Table S4). Additionally, we found two fragments with homology to the proteins of DNA viruses (*Cressdnaviricota* and *Cirlivirales*).

## 4. Discussion

Millipedes constitute the third-largest class of terrestrial arthropods; they are especially diverse in tropical areas [15,16], but their virome remains largely unknown. Moreover, when millipedes are studied in large-scale metagenomic surveys, they are often not identified even to the genus level [1,2,64]. This situation creates gaps in our knowledge of virus biodiversity and can hinder further research. 

Here, we explored the virome of the millipedes collected in southern Vietnam. Fourteen species of millipede were studied, with thirteen identified to the species level and one identified to the genus level. Overall, our data indicate the presence of about 80 viruses; for 26 of them, complete or partial genomes were assembled. About a third of all the detected viruses were putative members of the order *Picornavirales*. This result is close to one obtained by Shi et al. in a large-scale metagenomic study, where around 28% of all viruses discovered in millipedes were putative members of the order *Picornavirales* [1]. 

It is usually hard to accurately determine whether the viruses detected in the study actually infect the object of the study, their symbionts and parasites, or components of the diet. However, assumptions can be made based on the translation table used by a virus, or the origin of phylogenetically close viruses. Some viruses, such as CTDV, CTPV, CTNV, CPXV, CPQV, and CHAV, were mostly grouped together with the viruses of other arthropods, and thus it is likely that they indeed infect millipedes.

CAsV and CAV are phylogenetically close (Figure 7A and Figure S3B) to typical representatives of the groups that infect only bacteria [69] and ascomycete fungi [56]. Thus, it is likely that CAsV and CAV do not infect millipedes themselves, but either some component of their diet or their symbionts or parasites. 

The situation is less clear in the case of CTZV. Zhaoviruses are believed to infect ciliates [1], based on the fact that most members need a ciliate translation table in order to form proper ORFs. Nevertheless, some viruses discovered recently, including CTZV, can be translated using a standard translation table (Figure 10A). Additionally, CTZV was grouped (although with low branch support) with Crogonang virus 90, another virus that can be translated using standard genetic code [66]. Thus, it is possible that CTZV infects *Thyropygus carli* itself, or at least some non-ciliate organism associated with this millipede. More research is required to enhance our understanding of the host range and evolution of zhaoviruses.

In other cases, millipede viruses could be confidently clustered with viruses detected in vertebrates. In particular, CHySV was grouped with viruses discovered in *Phylloscopus borealis* and *Sphenodon punctatus* cloaca swabs (Figure 2B). Both of these species are insectivorous, and it is possible that the viruses from cloaca swabs were millipede viruses (or viruses from other arthropods). Similarly, the Cat Tien Termitodesmus and Cat Tien Hylomus picorna-like viruses clustered with a virus discovered in the bird metagenome (Figure 2F). 

Some millipede viruses discovered both here (CASV, DASV, CHSV, Figure 2B; CPCV, Figure 9A) and during previous large-scale studies [1,64] clustered with viruses recently discovered in shrews [44]. Reads of picorna-like viruses in shrews were predominantly found in the gut [44]. Since the shrews were studied in pools, it is possible that those viruses originated from one or a few millipedes consumed by the shrews prior to capture. 

The situation is most interesting in the case of the Wufeng shrew chuvirus 1. This virus was found in both the spleens and guts of shrews in compatible amounts, and it was assumed to be a virus of shrews [44], but it forms a monophyletic group with CPCV and the millipede chuvirus. We can hypothesize that shrews might have acquired an ancestor of the Wufeng shrew chuvirus 1 from millipedes.

Overall, 10 viruses (38% of the viruses with a full or partial genome) found in our study were phylogenetically related to viruses discovered in other arthropods, and they can thus be assumed to be millipede viruses. It should be noted that 3 out of these 10 viruses were the most abundant in the study. For two viruses (CAsV and CAV) discovered in the study (8%), we can confidently say that they are highly likely not to infect millipedes. In all other cases, the data that are currently available are not sufficient to draw any conclusions regarding the origins of those viruses.

Where possible, we followed the ICTV guidelines for species and genus demarcation [4,5,53,56,69]. At least eight of the viruses discovered here meet the criteria to be recognized as new species. For the CPXV, the species and genus demarcation criteria are met, and even demarcation as a new family can be discussed. Additionally, for CTNV, its demarcation as a new genus should be considered. However, the final decision on nomenclature and classification of the abovementioned cases can only be made by the ICTV.

All of the complete and partial genomes recovered in this study were highly divergent from the viruses currently deposited in the GenBank database: the overall amino acid identity according to BLAST was 21–72%, with more than half of the viruses having 40% or less identity to their closest relative. This emphasizes the need to improve our knowledge of virus diversity in millipedes and other neglected groups of arthropods. In addition to enhancing our understanding of virus evolution, such data can strongly improve interpretations of large-scale virome studies of both invertebrates and vertebrates.

## Figures and Tables

**Table 1:** Millipede specimens used in the study and pool composition.

Pool	Specimens in the Pool	Species
1	4	*Antheromorpha festiva* (Brölemann, 1896)
2	4	*Atopochetus dollfusii* (Pocock, 1893)
3	6	*Cryxus ovalis* (Linnaeus, 1758)
4	1	*Helicorthomorpha* cf. *holstii* (Pocock, 1895)
5	5	*Hyleoglomeris cattienensis* (Golovatch and Semenyuk, 2016)
6	3	*Hylomus cattienensis* (Nguyen, Golovatch, and Anichkin, 2005)
7	4	*Hylomus pilosus* (Attems, 1937)
8	4	*Nedyopus dawydoffiae* (Attems, 1953)
9	13	*Orthomorpha rotundicollis* (Attems, 1937)
10	5	*Plusioglyphiulus ampullifer* (Golovatch, Geoffroy, Mauriès, and VandenSpiegel, 2009)
11	4	*Thyropygus carli* (Attems, 1938)
12	1	*Touranella moniliformis* (Golovatch and Semenyuk, 2018)
13	1	*Termitodesmus* sp.
14	1	*Trigoniulus corallinus* (Gervais, 1842)

## Data Availability

Raw high-throughput sequencing data obtained during this study are available in the SRA database (BioProject accession number PRJNA1138258). Obtained virus sequences were deposited in the GenBank database (accession numbers PQ106591-PQ106644; PQ106731-PQ106771).

## Supplementary Materials

The following supporting information can be downloaded at: https://www.mdpi.com/article/10.3390/v16091486/s1, Table S1: Detailed information on millipede homogenization; Table S2: Detailed information on the construction of each phylogenetic tree; Table S3: Complete and partial genomes assembled in the study; Table S4: Genome fragments detected in the study; Table S5: Viruses with the highest abundance in the study; Table S6: Blastp results for ORFs in virus-like sequences; Figure S1: Examples of millipede specimens used in the study; Figure S2: Genomic structure and phylogenetic relationships of phasma-like viruses detected in the study; Figure S3: Genomic structure and phylogenetic relationships of the Cat Tien ashcevirus; Figure S4: Genomic structure and phylogenetic relationships of the Cat Tien Hylomus solemo-like virus; Figure S5: Genomic structure and phylogenetic relationships of the Cat Tien Trigoniulus hepe-like virus.
