# Supplementary material for: Extensive Diversity of Viruses in Millipedes Collected in the Dong Nai Biosphere Reserve (Vietnam)
Source: Viruses. 2024 Sep 19;16(9):1486. doi: 10.3390/v16091486 (PMC11437466; doi:10.3390/v16091486)
Supplement: Supplementary file 1 [file viruses-16-01486-s001.zip › viruses-3168466-supplementary.pdf]

Table S1. Detailed information on millipede homogenization

Specimen ID	Species	Collection date	The amount of saline buffer used for homogenization (µl)	
			Head	1 cm body part
36850	*Thyropygus carli*	28.11.2022	200	300
36851	*Helicorthomorpha cf. holstii*	27.11.2022	150	200
36852	*Atopochetus dollfusii*	27.11.2022	150	300
36853	*Touranella moniliformis*	04.12.2022	150	150
36854	*Nedyopus dawydoffiae*	02.12.2022	200	500
36855	*Trigoniulus corallinus*	06.12.2022	200	500
36856/1	*Hyleoglomeris cattienensis*	08.12.2022	200	**200***
36856/2	*Hyleoglomeris cattienensis*	08.12.2022	200	**300***
36856/3	*Hyleoglomeris cattienensis*	08.12.2022	200	**300***
36856/4	*Hyleoglomeris cattienensis*	08.12.2022	200	**300***
36856/5	*Hyleoglomeris cattienensis*	08.12.2022	150	**300***
36857/1	*Nedyopus dawydoffiae*	08.12.2022	200	300
36857/2	*Nedyopus dawydoffiae*	08.12.2022	200	300
36857/3	*Nedyopus dawydoffiae*	08.12.2022	200	300
36858/1	*Plusioglyphiulus ampullifer*	08.12.2022	150	300
36858/2	*Plusioglyphiulus ampullifer*	08.12.2022	150	300
36858/3	*Plusioglyphiulus ampullifer*	08.12.2022	150	300
36858/4	*Plusioglyphiulus ampullifer*	08.12.2022	150	300
36859/1	*Orthomorpha rotundicollis*	08.12.2022	300	500
36859/2	*Orthomorpha rotundicollis*	08.12.2022	150	200
36859/3	*Orthomorpha rotundicollis*	08.12.2022	150	300
36859/4	*Orthomorpha rotundicollis*	08.12.2022	150	200
36859/5	*Orthomorpha rotundicollis*	08.12.2022	150	200
36859/6	*Orthomorpha rotundicollis*	08.12.2022	150	200
36859/7	*Orthomorpha rotundicollis*	08.12.2022	150	300
36860/1	*Hylomus pilosus*	08.12.2022	200	500
36860/2	*Hylomus pilosus*	08.12.2022	200	500
36860/3	*Hylomus pilosus*	08.12.2022	150	300
36860/4	*Hylomus pilosus*	08.12.2022	150	300
36861/1	*Hylomus cattienensis*	08.12.2022	200	600
36861/2	*Hylomus cattienensis*	08.12.2022	200	600
36861/3	*Hylomus cattienensis*	08.12.2022	200	600
36862/1	*Cryxus ovalis*	09.12.2022	150	200
36862/2	*Cryxus ovalis*	09.12.2022	200	300
36862/3	*Cryxus ovalis*	09.12.2022	150	300
36862/4	*Cryxus ovalis*	09.12.2022	150	200
36862/5	*Cryxus ovalis*	09.12.2022	150	200
36862/6	*Cryxus ovalis*	09.12.2022	150	200
36863	*Thyropygus carli*	04.12.2022	400	500
36864	*Plusioglyphiulus ampullifer*	07.12.2022	150	200
36865/1	*Orthomorpha rotundicollis*	07.12.2022	200	500
36865/2	*Orthomorpha rotundicollis*	07.12.2022	150	200
36866	*Antheromorpha festiva*	07.12.2022	150	200
36867	*Termitodesmus sp.*	07.12.2022	150	200
36868	*Thyropygus carli*	07.12.2022	400	500
36869	*Thyropygus carli*	07.12.2022	400	750
36870/1	*Atopochetus dollfusii*	07.12.2022	300	500
36870/2	*Atopochetus dollfusii*	07.12.2022	200	500
36870/3	*Atopochetus dollfusii*	07.12.2022	200	500

36871/1	*Orthomorpha rotundicollis*	07.12.2022	200	500
36871/2	*Orthomorpha rotundicollis*	07.12.2022	200	500
36871/3	*Orthomorpha rotundicollis*	07.12.2022	200	500
36871/4	*Orthomorpha rotundicollis*	07.12.2022	200	500
36872/1	*Antheromorpha festiva*	07.12.2022	200	300
36872/2	*Antheromorpha festiva*	07.12.2022	150	200
36872/3	*Antheromorpha festiva*	07.12.2022	150	200

* - whole body was homogenized instead of 1cm body part

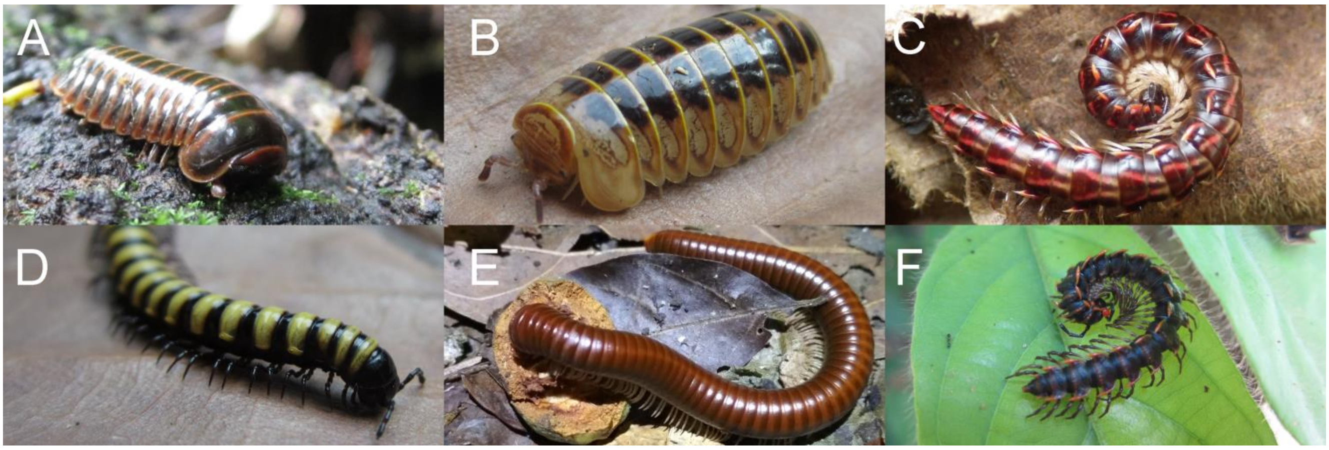

Figure S1. Examples of millipede specimens used in the study. (A) *Cryxus ovalis* (B) *Hyleoglomeris cattienensis* (C) *Antheromorpha festiva* (D) *Nedyopus dawydoffiae* (E) *Thyropygus carli* (F) *Orthomorpha rotundicollis*. Photo by Irina I. Semenyuk.

Table S2. Detailed information on the construction of each phylogenetic tree.

Tree	Associated Figure	Protein used	Number of sequences	Final alignment length	IQ-TREE model
Picornavirales and related viruses	Figure 2	polyprotein containing RdRp domains	191	1014	Q.pfam+F+I+R8
Tolivirales, Nodamuvirales and related viruses	Figure 3	RdRp-containing protein	124	318	Q.pfam+F+R6
Nairoviridae and related viruses	Figure 4	L	29	2861	Q.yeast+F+R5
Xinmoviridae Lispiviridae Bornaviridae Nyamiviridae and related viruses	Figure 5	L	49	1367	Q.pfam+F+I+R5
Artiviridae	Figure 6	VP2	27	620	LG+I+G4
Alternaviridae	Figure 7	RdRp-containing protein	23	919	LG+F+R4
Unuamitovirus	Figure 8	RdRp	66	490	Q.pfam+F+I+R5
Chuviridae	Figure 9	L	51	1743	LG+F+I+R6
zhao-like viruses	Figure 10	RdRp	14	312	LG+R3
Qinviridae	Figure 11	L	27	1133	Q.pfam+F+R4
Phasma-like	Figure S2A	L	70	1630	Q.pfam+F+I+R6
Phasma-like	Figure S2B	G	26	1126	WAG+F+I+R3
Ashcevirus	Figure S3	RdRp	26	502	LG+F+I+G4
Solemo-like	Figure S4	VP2	46	269	LG+I+G4
Hepe-like	Figure S5	Polyprotein	35	777	Q.pfam+F+I+R4

Table S3. Complete and partial genomes assembled in the study.

Virus group	Millipede species	Virus name	Closest relative in GenBank	Protein	Query cover	Identity
Picornavirales	*Atopochetus dollfusii*	**Cat Tien Atopochetus seco-like virus***	[WPV63522.1] Wufeng shrew picorna-like virus 10	Polyprotein	31%	27%
	Atopochetus dollfusii	**Dong Nai Atopochetus seco-like virus**	[WPV63585.1] Wufeng shrew picorna-like virus 46	Polyprotein	19%	44%
	Hyleoglomeris cattienensis	Cat Tien Hyleoglomeris seco-like virus	[WEM04567.1] Riboviria sp.	Polyprotein	85%	50%
	Hylomus cattienensis	**Cat Tien Hylomus picorna-like virus**	[WKV34040.1] Riboviria sp.	RdRp	87%	44%
	Hylomus pilosus	**Cat Tien Hylomus seco-like virus**	[WPV63585.1] Wufeng shrew picorna-like virus 46	Polyprotein	23%	60%
	Termitodesmus sp.	**Cat Tien Thermitodesmus picorna-like virus**	[WKV33537.1] Riboviria sp.	RdRp	43%	34%
	Trigoniulus corallinus	**Cat Tien Trigoniulus dicistro-like virus**	[AWK77854.1] Bundaberg bee virus 1	ORF1	96%	39%
Cryppavirales	*Hylomus pilosus*	**Cat Tien Hylomus unuamitovirus**	[QDH90007.1] Mitovirus sp.	RdRp	95%	33%
	Hylomus pilosus	Dong Nai Hylomus unuamitovirus	[QDH90007.1] Mitovirus sp.	RdRp	97%	38%
	Hylomus pilosus	Da Huoai Hylomus unuamitovirus	[UJQ92580.1] Mitoviridae sp.	RdRp	89%	51%
Bunyaviricetes	*Cryxus ovalis*	Cat Tien Cryxus phasma-like virus	[QKN84397.1] Rice Phasma-like virus 1	RdRp	73%	25%
	Thyropygus carli	**Cat Tien Thyropygus nairo-like virus**	[YP_009293594.1] Sanxia Water Strider Virus 1	RdRp	93%	29%
	Trigoniulus corallinus	Cat Tien Trigoniulus phasma-like virus	[APG79334.1] Hubei bunya-like virus 11	RdRp	15%	26%
Tolivirales	*Cryxus ovalis*	**Cat Tien Cryxus carmotetra-like virus**	[WOK58419.1] Wufeng shrew carmotetravirus 1	RdRp	88%	46%
Nodamuvirales	*Thyropygus carli*	Cat Tien Thyropygus noda-like virus	[YP_009337883.1] Hubei orthoptera virus 4	RdRp	91%	61%
	Trigoniulus corallinus	Cat Tien Trigoniulus noda-like virus	[UQB76086.1] Flumine nodavirus 16	RdRp	88%	54%
Kitrinoviricota	*Thyropygus carli*	**Cat Tien Thyropygus kitrino-like virus**	[WKV33084.1] Riboviria sp.	RdRp	90%	21%
Ghabrivirales	*Atopochetus dollfusii*	**Cat Tien alterna-like virus**	[YP_007353985.1] Aspergillus foetidus dsRNA mycovirus	RdRp	96%	38%
	Hyleoglomeris cattienensis	**Cat Tien Hyleoglomeris arti-like virus**	[WZH61638.1] Sonukit virus	RdRp	70%	38%

Leviviricetes	*Hylomus cattienensis*	Cat Tien ashcevirus	[YP_010770350] ssRNA phage SRR7976310_17	RdRp	98%	53%
Sobelivirales	*Hylomus pilosus*	Cat Tien Hylomus solemo-like virus	[YP_009330046.1] Hubei sobemo-like virus 16	RdRp	35%	72%
Jingchuvirales	*Plusioglyphiulus ampullifer*	**Cat Tien Plusioglyphiulus chu-like virus**	[WPR16559.1] Millipede chuvirus	RdRp	99%	45%
Muvirales	*Plusioglyphiulus ampullifer*	**Cat Tien Plusioglyphiulus qin-like virus**	[YP_009337847.1] Hubei qinvirus-like virus 1	RdRp	84%	35%
Mononegavirales	*Plusioglyphiulus ampullifer*	**Cat Tien Plusioglyphiulus xinmo-like virus**	[YP_010800574.1] Odonatan anphe-related virus OKIAV59	RdRp	84%	27%
Zhaoviruses	*Thyropygus carli*	**Cat Tien Thyropygus zhao-like virus**	[YP_009337419.1] Beihai zhaovirus-like virus 1	Polyprotein	38%	29%
Hepeviridae	*Trigoniulus corallinus*	Cat Tien Trigoniulus hepe-like virus	[WDS50647.1] Riboviria sp.	RdRp	36%	26%

* - viruses with complete coding genome are in bold

Table S4. Genome fragments detected in the study.

Pool number and millipede species	Contigs group	Number of contigs in the group	Largest contig length (nt)	Closest relative in GenBank (for largest contig, according to protein blast)	Query cover	Identity
№1 - *Antheromorpha festiva*	durnavirales	1	935	[AVA30703.1] RNA-dependent RNA polymerase [Partitiviridae sp.]	100%	61%
	picornavirales	6	826	[QJI53518.1] hypothetical protein 2 [Picornavirales sp.]	96%	41%
	tymovirales	5	845	[QYF50213.1] hypothetical protein 2 [Xinjiang deltaflexi-like virus 1]	100%	86%
	nodamuvirales	1	612	[QIC52852.1] capsid protein [Apple virus C]	98%	42%
№2 - *Atopochetus dollfusii*	ghabrivirales	1	692	[UYL95304.1] 127 kDa protein [Baoding Chrys tick virus 1]	97%	51%
	polymycoviridae	2	580	[YP_009352876.1] Beauveria bassiana polymycovirus 1	97%	55%
	leviviricetes	4	920	[YP_010769241.1] ssRNA phage Esthiorhiza.2_49	93%	57%
	elliovirales	1	1683	[KAG8175021.1] Oedothorax gibbosus	91%	31%
	picornavirales	5	656	[USC27682.1] RNA dependent RNA polymerase [Picornaviridae sp. gcode 4]	100%	100%
№ 3 - *Cryxus ovalis*	leviviricetes	1	452	[QDH91163.1] RNA-dependent RNA polymerase [Leviviridae sp.]	84%	52%
	picornavirales	1	419	[UXD80012.1] putative polyprotein [Myrmica rubra picorna-like virus 3]	100%	99%
№4 - *Helicorthomorpha cf. holstii*	nothing					
№5 - *Hyleoglomeris cattienensis*	tolivirales	6	3182	[WKV33281.1] RNA-dependent RNA polymerase [Riboviria sp.]	71%	39%
№6 *Hylomus cattienensis*	picornavirales a	3	6959	[WPV63207.1] RNA-dependent RNA polymerase [Wufeng shrew dicistrovirus 12]	99%	65%
	picornavirales b	4	526	non-structural polyprotein [PNG bee virus 4]	97%	65%
	durnavirales	1	439	[UBR58449.1]capsid protein [Rhizoctonia solani partitivirus 11]	99%	74%
	quenyaviruses	1	454	[QFR59045.1] putative RNA dependent RNA polymerase [Hongshan virus]	98%	55%
	leviviricetes	3	1029	[APG77225.1] hypothetical protein [Hubei levi-like virus 5]	99	54%
№7 - *Hylomus pilosus*	durnavirales	4	772	[WKV33317.1] RNA-dependent RNA polymerase [Riboviria sp.]	73%	59%
	leviviricetes	1	1334	[YP_010769641.1] RNA-directed RNA polymerase [ssRNA phage SRR6960799_24]	95%	41%
	cryppavirales	6	1263	[WLK77420.1] RNA-dependent RNA polymerase [Suillus luteus mitovirus 3]	97%	39%
	martellivirales	5	483	[YP_009362088.1] protein 126 [Hoya chlorotic spot virus]	100%	67%
	picornavirales a	9	986	[QKW94216.1] calicivirus coat protein [PNG bee virus 12]	97%	25%
	picornavirales b	8	1125	[UNY42104.1] polyprotein 2 [Picornavirales sp.]	97%	49%
	picornavirales c	1	471	[WIW43231.1] putative polyprotein [Pteropus rufus picorna-like virus 1]	100%	69%
№8 - *Nedyopus dawydoffiae*	reovirales	2	461	[UPT53743.1]hypothetical protein 1 [Bactrocera dorsalis orbivirus isolate Bt]	98%	70%
№9 - *Orthomorpha rotundicollis*	picornavirales a	12	2161	[QJI53517.1] hypothetical protein 1 [Picornavirales sp.]	46%	28%
	martellivirales	1	899	[YP_009333242.1]RdRp [Beihai charybdis crab virus 1]	93%	37%
	nodamuvirales	5	868	[UNI73861.1] RNA-dependent RNA polymerase [brine shrimp noda-like virus 1]	100%	54%
	picornavirales b	3	636	[WPV63436.1] Longquan bat picorna-like virus 5	100%	76%

	picornavirales c	2	580	[UCR92493.1] polyprotein [Apis picorna-like virus 4]	100%	95%
№10 - *Plusioglyphiulus ampullifer*	picornavirales a	3	1340	[YP_009336567.1] hypothetical protein [Hubei picorna-like virus 49]	83%	32%
	tolivirales	1	533	[UYL83183.1] RNA-dependent RNA polymerase [XiangYun tombus-noda-like virus 5]	100%	61%
	picornavirales b	2	404	[UHK03210.1] polyprotein [Hangzhou dicistro-like virus 2]	48%	54%
№11 - *Thyropygus carli*	ghabrivirales	3	385	[UUT40381.1] RNA-dependent RNA polymerase [Geotrichum candidum totivirus 3b]	100%	99%
	cryppavirales	4	996	[AZJ25097.1] RNA-dependent RNA polymerase [Rhizophagus diaphanum mitovirus 4]	98%	56%
	cirlivirales	1	517	[YP_009506319.1]replication-association protein [Cyclovirus PK5222]	99%	52%
	tymovirales	1	419	[UZG77120.1] replicase [Cowpea mild mottle virus]	100%	77%
	picornavirales a	6	4861	[ULF50554.1] structural protein [Aparavirus sp.]	99%	99%
	durnavirales	1	389	[WPA70736.1] Mystacina tuberculata picobirnavirus 8	100%	67%
	picornavirales b	10	2181	[WPV63549.1] capsid protein [Wufeng shrew picorna-like virus 23]	89%	68%
№12 - *Touranella moniliformis*	nothing					
№13 - *Termitodesmus* sp.	piccovirales	3	498	[ARV85896.1] structural protein [Planococcus citri densovirus]	98%	55%
	picornavirales	3	2757	[USL85467.1] Avian associated calicivirus 1	96%	29%
	muvirales	4	1629	[YP_009337847.1] RNA-dependent RNA polymerase [Hubei qinvirus-like virus 1]	100%	38%
№14 - *Trigoniulus corallinus*	leviviricetes	1	462	[URG16438.1] RNA dependent RNA polymerase [Leviviridae sp.]	100%	70%
	wolframvirales	2	917	[UJQ92812.1] putative RNA-dependent RNA polymerase [Narnaviridae sp.]	92%	38%
	durnavirales	4	1240	[WWV90581.1] hypothetical protein [Riboviria sp.]	95%	43%
	picornavirales a	2	867	RNA-dependent RNA polymerase [Riboviria sp.]	83%	29%
	tolivirales	1	458	[WEU70900.1] RNA-dependent RNA polymerase [Hangzhou tombus-like virus 1]	99%	42%
	nodamuvirales	1	2227	[QKE55019.1] Picornaviridae sp.	54%	38%
	cressdnaviricota	1	1399	[QKN88897.1] capsid protein [Cressdnaviricota sp.]	98%	38%
	picornavirales b	1	397	[UXB54241.1] polyprotein [Anthonomus grandis iflavirus 1]	97%	63%
	picornavirales c	1	497	[WKV34372.1] RNA-dependent RNA polymerase [Riboviria sp.]	92%	37%
	picornavirales d	13	1138	[URG15003.1] RNA dependent RNA polymerase [Picornaviridae sp.]	94%	83%
	picornavirales e	4	958	[APS85749.1] non structural polyprotein [Biomphalaria virus 1]	83%	41%

Table S5. Viruses with the highest abundance in the study.

Pool number	Virus name	Abundance (reads % out of total reads after filtering)
2	Dong Nai Atopochetus seco-like virus	0.64%
11	Cat Tien Thyropygus nairo-like virus	3.73%
14	Cat Tien Trigoniulus hepe-like virus	0.33%
14	Cat Tien Trigoniulus phasma-like virus	2.52%

Table S6. Blastp results for ORFs in virus-like sequences.

Pool number	Name	ORF	ORF length (aa)	Closest relative in GenBank	Query cover	Identity	E-value
№5 - *Hyleoglomeris cattienensis*	Hyleoglomeris Partiti-like element	1	1062	[HEX5716198.1] Thermoanaerobaculia bacterium – **TPA: sulfotransferase**	9%	33.0%	0.009
		2	961	[QZZ63408.1] Nelson Partiti-like virus 1 – **putative RdRp**	17%	28.4%	4×10^{-7}
		3	915	no aa homology found in GenBank	-	-	-
		4	295	[YP_529731.1] Agrotis segetum nucleopolyhedrovirus A – **ORF-61**	38%	34.8%	0.001
	Hyleoglomeris Picorna-like element	1	575	no aa homology found in GenBank	-	-	-
		2	235	[WGZ60546.1] Picornaviridae sp. - **polyprotein**	30%	39.2%	8×10^{-4}
		3	235	no aa homology found in GenBank	-	-	-
		4	242	no aa homology found in GenBank	-	-	-
		5	2230	[QKN88967.1] Picornavirales sp. – **polyprotein (RdRp)**	34%	30.9%	9×10^{-95}
№7 - *Hylomus pilosus*	Hylomus astro-like element	1	658	[XP_023241119.1] Centruroides sculpturatus - **lipid storage droplets surface-binding protein 2-like**	54%	31.6%	3×10^{-44}
		2	169	[KAK3603511.1] Potamilus streckersoni - **hypothetical protein**	98%	34.9%	5×10^{-12}
		3	352	[YP_009330020.1] Hubei astro-like virus - **capsid protein**	86%	33.12%	10^{-34}
№10 - *Plusioglyphiulus ampullifer*	Plusioglyphiulus xinmo-like element	1	104	no aa homology found in GenBank	-	-	-
		2	406	[WPR16619.1] Pseudoscorpian xinmovirus - **RdRp**	90%	35%	10^{-60}
		3	306	[WPR16619.1] Pseudoscorpian xinmovirus - **RdRp**	99%	52%	6×10^{-96}
№13 - *Termitodesmus* sp.	Termitodesmus phasma-like element	1	188	[WPR17618.1] - Millipede phasma-like virus 2 - **glycoprotein**	95%	49.7%	9×10^{-61}
		2	132	[XP_043189829.1] Amphibalanus amphitrite - **uncharacterized protein**	99%	38%	4×10^{-22}
№14 - *Trigoniulus corallinus*	Trigoniulus phasma-like element	1	120	no aa homology found in GenBank	-	-	-
		2	199	[WPR17617.1] Millipede phasma-like virus - **glycoprotein**	74%	46.9%	$\mathbf{6\times10^{-51}}$
		3	81	no aa homology found in GenBank	-	-	-

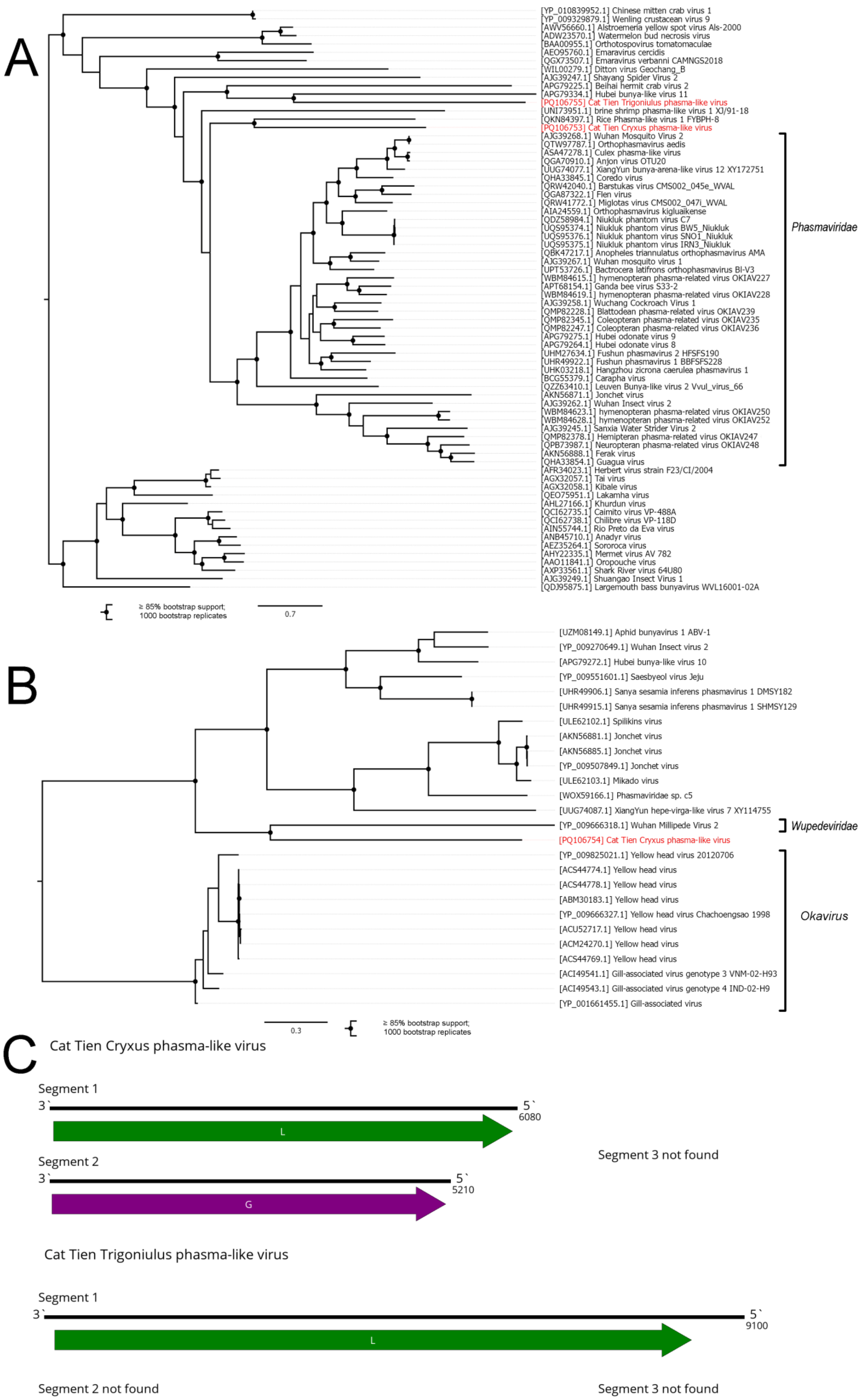

A
[YP_010839952.1] Chinese mitten crab virus 1
[YP_009329879.1] Wenling crustacean virus 9
[AWV56660.1] Alstroemeria yellow spot virus Als-2000
[ADW23570.1] Watermelon bud necrosis virus
[BAA00955.1] Orthotospovirus tomatomaculae
[AEO95760.1] Emaravirus cercidis
[QGX73507.1] Emaravirus verbanni CAMNGS2018
[WIL00279.1] Ditton virus Geochang_B
[AJG39247.1] Shayang Spider Virus 2
[APG79225.1] Beihai hermit crab virus 2
[APG79334.1] Hubei bunya-like virus 11
[PQ106755] Cat Tien Trigoniulus phasma-like virus
[UNI73951.1] brine shrimp phasma-like virus 1 XJ/91-18
[QKN84397.1] Rice Phasma-like virus 1 FYBPH-8
[PQ106753] Cat Tien Cryxus phasma-like virus
[AJG39268.1] Wuhan Mosquito Virus 2
[QTW97787.1] Orthophasmavirus aedis
[ASA47278.1] Culex phasma-like virus
[QGA70910.1] Anjon virus OTU20
[UUG74077.1] XiangYun bunya-arena-like virus 12 XY172751
[QHA33845.1] Coredo virus
[QRW42040.1] Barstukas virus CMS002_045e_WVAL
[QGA87322.1] Flen virus
[QRW41772.1] Miglotas virus CMS002_047i_WVAL
[AIA24559.1] Orthophasmavirus kigluaikense
[QDZ58984.1] Niukluk phantom virus C7
[UQS95374.1] Niukluk phantom virus BW5_Niukluk
[UQS95376.1] Niukluk phantom virus SNO1_Niukluk
[UQS95375.1] Niukluk phantom virus IRN3_Niukluk
[QBK47217.1] Anopheles triannulatus orthophasmavirus AMA
[AJG39267.1] Wuhan mosquito virus 1
[UPT53726.1] Bactrocera latifrons orthophasmavirus Bl-V3
[WBM84615.1] hymenopteran phasma-related virus OKIAV227
[APT68154.1] Ganda bee virus S33-2
[WBM84619.1] hymenopteran phasma-related virus OKIAV228
[AJG39258.1] Wuchang Cockroach Virus 1
[QMP82228.1] Blattodean phasma-related virus OKIAV239
[QMP82345.1] Coleopteran phasma-related virus OKIAV235
[QMP82247.1] Coleopteran phasma-related virus OKIAV236
[APG79275.1] Hubei odonate virus 9
[APG79264.1] Hubei odonate virus 8
[UHM27634.1] Fushun phasmavirus 2 HFSFS190
[UHR49922.1] Fushun phasmavirus 1 BBFSFS228
[UHK03218.1] Hangzhou zicrona caerulea phasmavirus 1
[BCG55379.1] Carapha virus
[QZZ63410.1] Leuven Bunya-like virus 2 Vvul_virus_66
[AKN56871.1] Jonchet virus
[AJG39262.1] Wuhan Insect virus 2
[WBM84623.1] hymenopteran phasma-related virus OKIAV250
[WBM84628.1] hymenopteran phasma-related virus OKIAV252
[AJG39245.1] Sanxia Water Strider Virus 2
[QMP82378.1] Hemipteran phasma-related virus OKIAV247
[QPB73987.1] Neuropteran phasma-related virus OKIAV248
[AKN56888.1] Ferak virus
[QHA33854.1] Guagua virus
Phasmaviridae
[AFR34023.1] Herbert virus strain F23/CI/2004
[AGX32057.1] Tai virus
[AGX32058.1] Kibale virus
[QEO75951.1] Lakamha virus
[AHL27166.1] Khurdun virus
[QCI62735.1] Caimito virus VP-488A
[QCI62738.1] Chilibre virus VP-118D
[AIN55744.1] Rio Preto da Eva virus
[ANB45710.1] Anadyr virus
[AEZ35264.1] Sororoca virus
[AHY22335.1] Mermet virus AV 782
[AAO11841.1] Oropouche virus
[AXP33561.1] Shark River virus 64U80
[AJG39249.1] Shuangao Insect Virus 1
[QDJ95875.1] Largemouth bass bunyavirus WVL16001-02A
≥ 85% bootstrap support;
1000 bootstrap replicates
0.7
B
[UZM08149.1] Aphid bunyavirus 1 ABV-1
[YP_009270649.1] Wuhan Insect virus 2
[APG79272.1] Hubei bunya-like virus 10
[YP_009551601.1] Saesbyeol virus Jeju
[UHR49906.1] Sanya sesamia inferens phasmavirus 1 DMSY182
[UHR49915.1] Sanya sesamia inferens phasmavirus 1 SHMSY129
[ULE62102.1] Spilikins virus
[AKN56881.1] Jonchet virus
[AKN56885.1] Jonchet virus
[YP_009507849.1] Jonchet virus
[ULE62103.1] Mikado virus
[WOX59166.1] Phasmaviridae sp. c5
[UUG74087.1] XiangYun hepe-virga-like virus 7 XY114755
[YP_009666318.1] Wuhan Millipede Virus 2
Wupedeviridae
[PQ106754] Cat Tien Cryxus phasma-like virus
[YP_009825021.1] Yellow head virus 20120706
[ACS44774.1] Yellow head virus
[ACS44778.1] Yellow head virus
[ABM30183.1] Yellow head virus
[YP_009666327.1] Yellow head virus Chachoengsao 1998
[ACU52717.1] Yellow head virus
[ACM24270.1] Yellow head virus
[ACS44769.1] Yellow head virus
[ACI49541.1] Gill-associated virus genotype 3 VNM-02-H93
[ACI49543.1] Gill-associated virus genotype 4 IND-02-H9
[YP_001661455.1] Gill-associated virus
Okavirus
0.3
≥ 85% bootstrap support;
1000 bootstrap replicates
C
Cat Tien Cryxus phasma-like virus
Segment 1
3`
5`
6080
L
Segment 3 not found
Segment 2
3`
5`
5210
G
Cat Tien Trigoniulus phasma-like virus
Segment 1
3`
5`
9100
L
Segment 2 not found
Segment 3 not found

Figure S2. Genomic structure and phylogenetic relationships of phasma-like viruses detected in the study. (A)Phylogenetic tree, constructed using the amino acid sequences of the RdRp, and rooted on the family *Peribunyaviridae*. (B) Phylogenetic tree, constructed using the amino acid sequences ofG protein, rooted on the genus *Okavirus*. The scale bar represents the number of amino acid substitutions per site. The discovered viruses are marked in red. (C) Genome scheme of the phasma-like viruses. The RdRp-encoding ORF is marked in green.

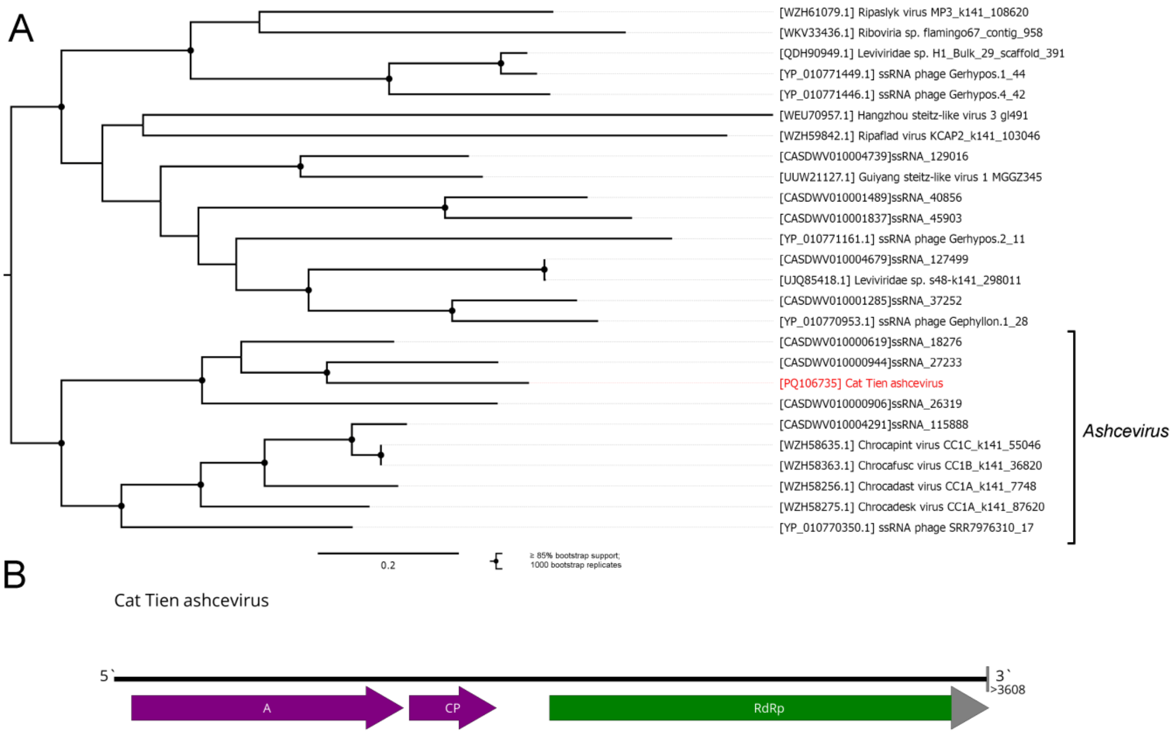

Figure S3. Genomic structure and phylogenetic relationships of the Cat Tien ashcevirus. (A) Phylogenetic tree of Cat Tien ashcevirus, constructed using amino acid sequences of the RdRp. Tree is rooted on genera *Nehumivirus* and *Endehruvirus*.The scale bar represents the number of amino acid substitutions per site. The discovered virus is marked in red. (B) Genome scheme of the Cat Tien ashcevirus. The RdRp-encoding ORF is marked in green. The gray line indicates an estimated gap. The gray arrowhead indicates absence of the stop-codon within the sequenced region.

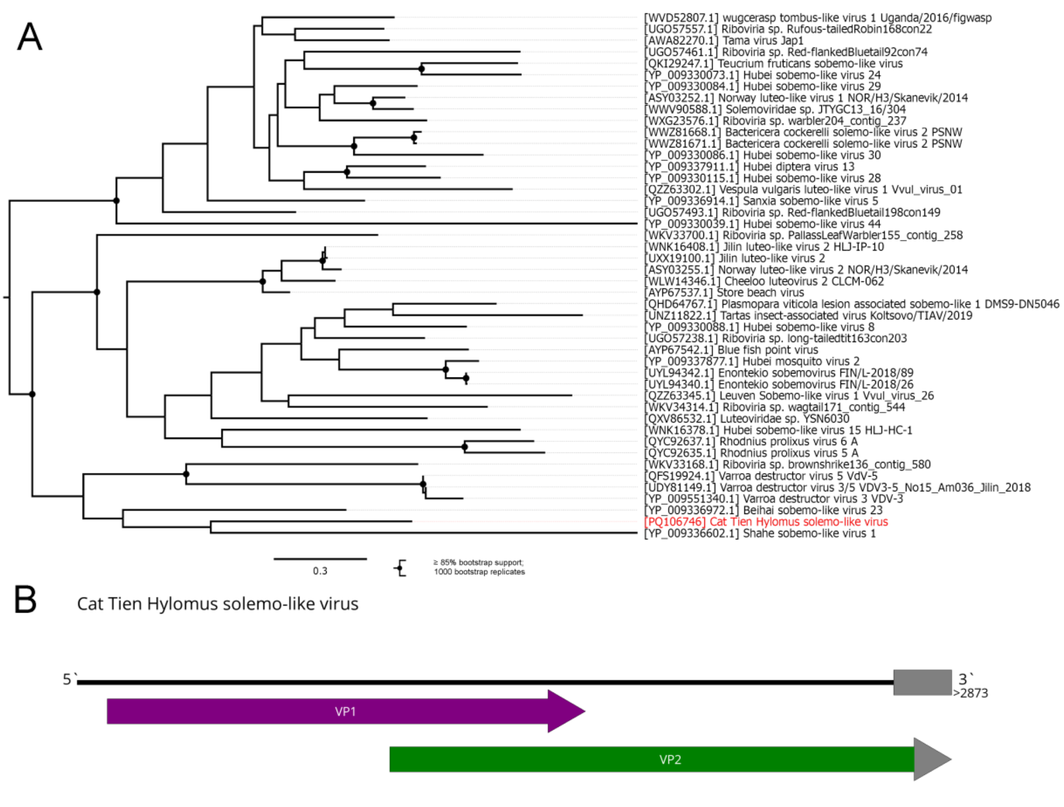

Figure S4. Genomic structure and phylogenetic relationships of the Cat Tien Hylomus solemo-like virus. (A) Midpoint-rooted phylogenetic tree of Cat Tien Hylomus solemo-like virus, constructed using the amino acid sequences of the RdRp. The scale bar represents the number of amino acid substitutions per site. The discovered virus is marked in red. (B) Genome scheme of the Cat Tien Hylomus solemo-like virus. The RdRp-encoding ORF is marked in green. The gray block indicates an estimated gap. The gray arrowhead indicates the absence of the stop-codon within the sequenced region.

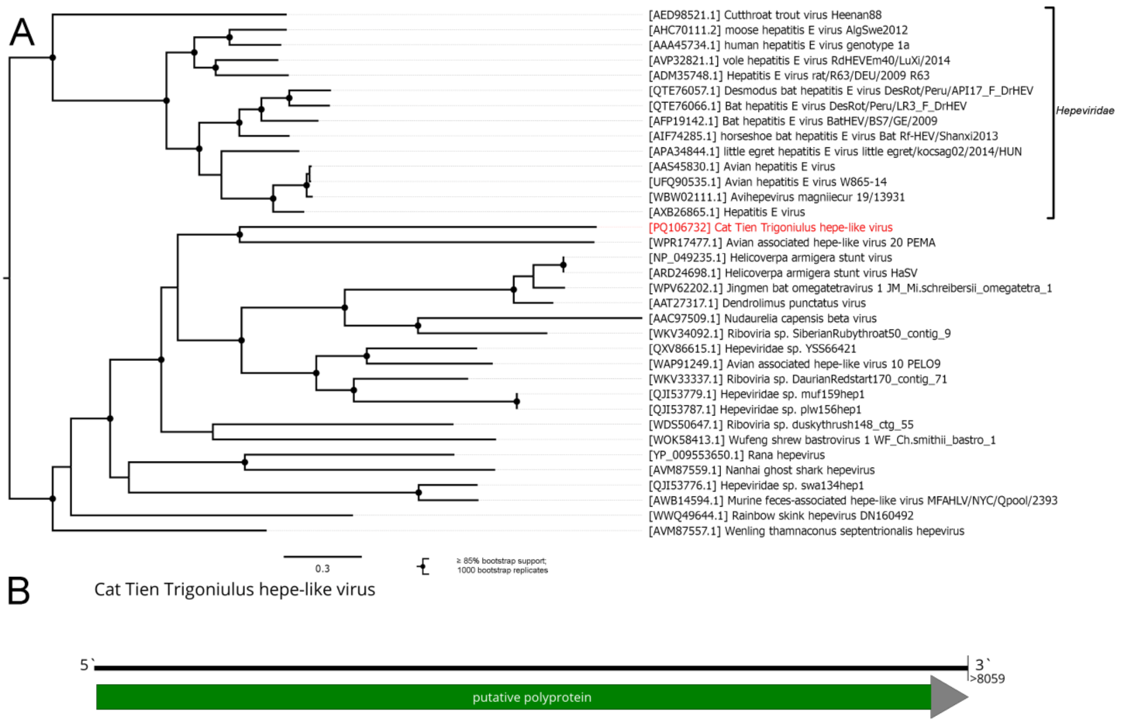

Figure S5. Genomic structure and phylogenetic relationships of the Cat Tien Trigoniulus hepe-like virus. (A) Phylogenetic tree of the Cat Tien Trigoniulus hepe-like virus, constructed using the amino acid sequences of the polyprotein. Tree is rooted on the family *Hepeviridae*. The scale bar represents the number of amino acid substitutions per site. The discovered virus is marked in red. (B) Genome scheme of the Cat Tien Trigoniulus hepe-like virus. The RdRp-encoding ORF is marked in green. The gray line indicates the estimated gap. The gray arrowhead indicates the absence of the stop-codon within the sequenced region.
